# Expression of ncRNAs on the DLK1-DIO3 Locus Is Associated With Basal and Mesenchymal Phenotype in Breast Epithelial Progenitor Cells

**DOI:** 10.3389/fcell.2020.00461

**Published:** 2020-06-16

**Authors:** Zuzana Budkova, Anna Karen Sigurdardottir, Eirikur Briem, Jon Thor Bergthorsson, Snævar Sigurdsson, Magnus Karl Magnusson, Gunnhildur Asta Traustadottir, Thorarinn Gudjonsson, Bylgja Hilmarsdottir

**Affiliations:** ^1^Stem Cell Research Unit, Biomedical Center, Department of Anatomy, Faculty of Medicine, School of Health Sciences, University of Iceland, Reykjavik, Iceland; ^2^Department of Laboratory Hematology, Landspitali – University Hospital, Reykjavik, Iceland; ^3^Department of Pharmacology and Toxicology, Faculty of Medicine, School of Health Sciences, University of Iceland, Reykjavik, Iceland; ^4^Department of Pathology, Landspitali – University Hospital, Reykjavik, Iceland

**Keywords:** DLK1-DIO3 locus, *MEG3*, ncRNAs, epithelial plasticity, breast progenitor cells

## Abstract

Epithelial-to-mesenchymal transition (EMT) and its reversed process mesenchymal-to-epithelial transition (MET) play a critical role in epithelial plasticity during development and cancer progression. Among important regulators of these cellular processes are non-coding RNAs (ncRNAs). The imprinted DLK1-DIO3 locus, containing numerous maternally expressed ncRNAs including the lncRNA maternally expressed gene 3 (*MEG3*) and a cluster of over 50 miRNAs, has been shown to be a modulator of stemness in embryonic stem cells and in cancer progression, potentially through the tumor suppressor role of *MEG3*. In this study we analyzed the expression pattern and functional role of ncRNAs from the DLK1-DIO3 locus in epithelial plasticity of the breast. We studied their expression in various cell types of breast tissue and revisit the role of the locus in EMT/MET using a breast epithelial progenitor cell line (D492) and its isogenic mesenchymal derivative (D492M). Marked upregulation of ncRNAs from the DLK1-DIO3 locus was seen after EMT induction in two cell line models of EMT. In addition, the expression of *MEG3* and the maternally expressed ncRNAs was higher in stromal cells compared to epithelial cell types in primary breast tissue. We also show that expression of *MEG3* is concomitant with the expression of the ncRNAs from the DLK1-DIO3 locus and its expression is therefore likely indicative of activation of all ncRNAs at the locus. *MEG3* expression is correlated with stromal markers in normal tissue and breast cancer tissue and negatively correlated with the survival of breast cancer patients in two different cohorts. Overexpression of *MEG3* using CRISPR activation in a breast epithelial cell line induced partial EMT and enriched for a basal-like phenotype. Conversely, knock down of *MEG3* using CRISPR inhibition in a mesenchymal cell line reduced the mesenchymal and basal-like phenotype of the cell line. In summary our study shows that maternally expressed ncRNAs are markers of EMT and suggests that *MEG3* is a novel regulator of EMT/MET in breast tissue. Nevertheless, further studies are needed to fully dissect the molecular pathways influenced by non-coding RNAs at the DLK1-DIO3 locus in breast tissue.

## Introduction

Breast cancer is the most common cancer in women and the second most common cancer overall (Ghoncheh et al., 2016). Despite major advances in diagnosis and treatment of cancer in recent years, metastasis and development of resistance to cancer therapies continues to be a challenge, causing over 90% of all cancer-related deaths (Ben-Jacob et al., 2012). A major contributing factor to metastasis and drug resistance is the heterogeneity and plasticity of the cells within tumors (Dagogo-Jack and Shaw, 2018). Epithelial-to-mesenchymal transition (EMT), is a developmental process that can be hijacked by cancer cells (Zeisberg and Kalluri, 2004; Moustakas and Heldin, 2007; Radisky et al., 2007). Generally, cells undergoing EMT, acquire increased migration and invasive properties and show increased resistance to apoptosis (Robson et al., 2006; Cao et al., 2016). Through these processes, EMT is considered a major mediator of phenotypic plasticity in cancer cells, metastatic formation and drug resistance (Mani et al., 2008; Scheel and Weinberg, 2012; Ansieau, 2013; Nieto et al., 2016; Lu and Kang, 2019). Recently, hybrid E/M (or partial EMT) cells have been shown to have even more metastatic and stem cell potential compared to the full epithelial or mesenchymal phenotype (Pastushenko et al., 2018). A reversed program, mesenchymal-to–epithelial transition (MET) is considered to facilitate colonization in secondary sites and reverse the plastic mesenchymal phenotype back to an epithelial state (Lu and Kang, 2019). This, however, is debated and further studies will increase our knowledge of the role of EMT/MET in cancer progression and metastasis.

EMT can be initiated through intrinsic factors such as expression of EMT related transcription factors (SNAI1, SNAI2, TWIST1, ZEB1, or ZEB2), cadherin switch from E-cadherin (CDH1) to N-cadherin (CDH2) or through epigenetic mechanisms. It can also be brought on by extrinsic factors derived from the microenvironment, such as secreted soluble factors: transforming growth factor-β (TGF-β), epidermal growth factor (EGF), fibroblast growth factors (FGFs), hepatocyte growth factor (HGF) or Wnt signaling factors (Moustakas and Heldin, 2007; Peinado et al., 2007; De Craene and Berx, 2013; Wang and Zhou, 2013; Williams et al., 2019).

Non-coding RNAs (ncRNAs) are among intrinsic regulators of EMT (Zaravinos, 2015). It is increasingly apparent that the ncRNAs are crucial in normal development and disease, but its mechanistic mode of action is largely unknown (Liz and Esteller, 2016). The two major classes of non-coding RNAs are long non-coding RNA (lncRNAs) and microRNA (miRNAs). Accumulating evidence suggests that lncRNAs function in a broad range of cellular processes such as cell growth, survival, migration, invasion and differentiation (Mercer et al., 2009; Sun et al., 2013; Di Gesualdo et al., 2014; Fatica and Bozzoni, 2014). LncRNAs are defined by the size of their transcripts and are longer than 200 nucleotides (nt), with no protein-coding function (Eades et al., 2014). Unlike microRNAs, lncRNAs are poorly conserved, but function in a regulatory network at the transcriptional, post-transcriptional, and translational level. miRNAs are 22 nt long RNA molecules that regulate expression post-transcriptionally primarily by binding to three prime untranslated region (3′UTR) of target genes (Bartel, 2009).

The imprinted DLK1-DIO3 locus located on chromosome 14 contains three paternally expressed protein-coding genes (*DLK1*, *RTL1*, *DIO3*) and numerous maternally expressed non-coding genes, including the lncRNA maternally expressed gene 3 (*MEG3*), and a cluster of over 50 miRNAs (Zhang et al., 2010; Dill and Naya, 2018; Baulina et al., 2019; Li et al., 2019).

The DLK1-DIO3 locus has been described as an important contributor to pluripotency and stemness in embryonic stem cells (ESCs) (Kaneko et al., 2014). It discriminates between mouse induced pluripotent stem cells (iPCS) and mouse ESCs, where genes from the locus were strongly repressed in iPSC clones compared to ES clones (Liu et al., 2010; Stadtfeld et al., 2010a). Furthermore, activation of maternally expressed genes from the locus is a strong indicator of the developmental potential of iPSC (Kang et al., 2009). miRNAs from the DLK1-DIO3 locus have been shown to promote pluripotency by inhibition of differentiation and stimulation of self-renewal in mouse ES cells (Moradi et al., 2017) and were found to be increased in tumor-originating cancer cells from lung adenocarcinoma (Valdmanis et al., 2015).

*MEG3* is a potential tumor suppressor gene in several cancer types, mainly through the observation that *MEG3* expression is lower in various tumor tissues compared with non-tumor tissues of the same origin (Sheng et al., 2014; Sun et al., 2014, 2016; Yin et al., 2015; Chak et al., 2017; Molina-Pinelo et al., 2018). The tumor suppressor role of *MEG3* is ascribed to stabilization of p53 with inhibition of proliferation and promotion of apoptosis (Zhang et al., 2003, 2010; Zhou et al., 2007; Wang et al., 2012; Sun et al., 2016).

*MEG3* was reported to positively regulate EMT in lung (Terashima et al., 2017) and ovarian (Mitra et al., 2017) cancer. Furthermore, *MEG3* has been shown to contribute to the development of osteosarcoma through increased migration, invasion and decreased apoptosis (Wang and Kong, 2018). Higher levels of *MEG3* were detected in plasma from colorectal cancer patients compared with non-cancerous controls (Liu et al., 2019).

D492 is a primary breast epithelial cell line, immortalized with the E6 and E7 oncogenes from the human papilloma virus 16 (Gudjonsson et al., 2002). Therefore, the p53 protein, which mediates the previously described tumor suppressor role of *MEG3*, is repressed in this cell line. D492 can generate both luminal and basal/myoepithelial cells in monolayer and 3D culture, expressing luminal or myoepithelial keratins such as keratin 19 and keratin 14, respectively. Furthermore, when D492 cells are co-cultured with endothelial cells, they, can generate spindle-shaped colonies with EMT phenotype. D492M (mesenchymal) was established from one such spindle-shaped colony (Sigurdsson et al., 2011). D492M is a phenotypically stable EMT cell line. It has lost epithelial markers such as keratins, E-cadherin and TP63, and gained expression of mesenchymal markers such as N-cadherin (Sigurdsson et al., 2011; Hilmarsdottir et al., 2015). D492M has acquired classical properties of cancer stem cells, such as increased CD44/CD24 ratio, anchorage independent growth, resistance to apoptosis and increased migration/invasion (Sigurdsson et al., 2011). D492 serves as a model for branching morphogenesis and together D492 and D492M represent a unique EMT model of isogenic cell lines with an epithelial and mesenchymal phenotype, respectively (Briem et al., 2019b). The ability of D492 to undergo mesenchymal transition upon endothelial stimulation makes it a valuable cell model to study EMT induced by extrinsic factors, although it is important to note that neither D492 nor D492M are tumorigenic in mice.

In this study, we describe a new role for the DLK1-DIO3 locus in EMT and phenotypic plasticity of breast cells. Following EMT in breast epithelial cell lines, expression of the ncRNAs at the DLK1-DIO3 locus was increased. In addition, *MEG3* was highly expressed in stromal cells in breast tissue and its expression correlated with decreased survival in breast cancer. Moreover, increased expression of the ncRNAs at the DLK1-DIO3 locus in a breast epithelial progenitor cell line promoted cellular plasticity and induced partial EMT. Collectively, our study provides a further understanding of the role of the DLK1-DIO3 locus in cellular phenotype of breast cells and might provide important insight into novel therapeutic targets aimed at overcoming heterogeneity and therapy resistance in breast cancer.

## Materials and Methods

### Cell Lines

Both D492 and D492M were cultured in H14 medium, as described previously (Gudjonsson et al., 2002; Sigurdsson et al., 2011) in flasks coated with collagen I (Advanced BioMFatrix, 5005-B). HEK-293T cell were cultured in Dulbecco’s Modified Eagle Medium (DMEM), high glucose, GlutaMAX (TM), pyruvate (Gibco, 31966), supplemented with 10% Fetal bovine serum (FBS), penicillin and streptomycin (Gibco, 15140-122). Primary Human umbilical vein endothelial cells (HUVECs) were obtained from Landspitali, University Hospital in Reykjavik, Iceland, (with informed consent, approved by Landspitali Ethical Committee No. 35/2013), cultured in Endothelial Growth Medium 2 (EGM2) media (Lonza, CC-3162) supplemented with growth factors and 5% FBS, further referred to as EGM5 medium as previously described (Sigurdsson et al., 2011). HMLE (Elenbaas et al., 2001) is epithelial progenitor cell line, from which was derived mesenchymal cell line HMLEmes after stable induction of EMT-TF (Mani et al., 2008). HMLE and HMLEmes were cultured in chemically defined HMLE media, containing DMEM/F12 with penicillin and streptomycin and growth factors Insulin (Sigma, I1882) 10 μg/ml, EGF (Peprotech, AF-100-15) 10 ng/ml, Hydrocortisone (Sigma, H0888) 500 ng/ml.

Primary human luminal-epithelial cells (LEP), myoepithelial cells (MEP), breast endothelial cells (BRENCs) and fibroblast were isolated from breast reduction mammoplasties (with informed consent, approved by the Icelandic National Bioethics Committee VSN-13-057) as previously described (Sigurdsson et al., 2011) and maintained in chemically defined medium 3 (CDM3) and chemically defined medium 4 (CDM4) as previously described (Pechoux et al., 1999; Ingthorsson et al., 2010). All cells were maintained in an incubator with 5% CO_2_ at 37°C.

### 3D Cultures/Mammosphere Assays

3D cultures were carried out in a 48-well plate format (Corning, 353078) in growth factor reduced reconstituted basement membrane rBM (further referred to as Matrigel, Corning, 354230). 5–10 × 10^3^ cells were seeded in 150 μl of Matrigel per well. Plate was incubated in 5% CO_2_ at 37°C for 15 min to solidify the Matrigel and then 300 μl of H14 media was added on top. The cells were grown for 3 weeks and pictures were taken on day 1, 7, 14, and 21. Cell culture media was changed three times per week. The colonies were quantified at day 14. The total number of cells was converted into percentage.

For co-culture experiments, 0.5 × 10^3^ of the epithelial cells were co-cultured with 1 × 10^5^ of endothelial cells (HUVECs) and were resuspended in 150 μl of Matrigel. Plate was incubated in 5% CO_2_ at 37°C for 15 min to solidify the Matrigel and then 300 μl EGM5 media was added on top. HUVECs cultured in Matrigel are viable, however, quiescent, having supporting role in the epithelial cells’ proliferation. The effect of *MEG3* was quantified by counting all colonies bigger than 100 μm.

### Total RNAseq and Analysis of the Data

The gene microarray expression analysis from D492 and D492M was published previously from our group by Sigurdsson and colleagues (Sigurdsson et al., 2011) and the total RNA-sequencing comparing D492 and D492M was published by Halldorsson and colleagues (Halldorsson et al., 2017).

The RNA was extracted using Tri-Reagent (Thermo Fisher Scientific, AM9738) from 5 replicates for each cell line. Whole Transcriptome Sequencing of D492M^KD–CTRL^ and D492M^KD–MEG3^ was performed in deCODE genetics (Reykjavik, Iceland). RNA sequencing reads were mapped to the reference genome (Ensembl primary assembly, version GRCh38) using STAR version 2.6.1 (Dobin et al., 2013). The program htseq-count (Anders et al., 2015) was used to quantify how many reads match each gene in an annotation file (Ensemble version GRCh38.96). The data from htseq-count was imported into R (R Development Core Team, 2015) and differential expression (DE) analysis on D492M^KD–CTRL^ vs D492^KD–MEG3^ was performed using DESeq2 (Love et al., 2014). Prior to DE analysis, genes with expression less than two reads were discarded. *P-*values were corrected for multiple testing using the false discovery rate (FDR) method. To compare gene expression from D492M^KD–CTRL^ vs D492M^KD–MEG3^ a volcano plot was generated. *P* value cut-off of 0.05 was applied. Volcano plot over all data (*p* < 0.05) was made in R using the EnhancedVolcano package from BioConductor. The top ten most upregulated and downregulated genes according log2 fold change were labeled. Gene Set Enrichment Analysis (GSEA) was applied to identify enrichment of gene signatures. Comparative analysis was investigated using the “Hallmark” database. The list of significantly expressed pathways is presented as a bar plot.

### Quantitative RT-PCR Analysis

Total RNA was extracted with Tri-Reagent (Thermo Fisher Scientific, AM9738). 1 μg of RNA of each sample was reverse transcribed into complementary DNA (cDNA), using Random Hexamers (Thermo Fisher Scientific, N8080127) and SuperScript IV Reverse Transcriptase (Thermo Fisher Scientific, 18090-200) kit and subjected to quantitative real time PCR (qRT-PCR) using Sybr Green dye Luna^®^ Universal qPCR Master Mix (NEB, M3003L) or TaqMan probes Luna^®^ Universal Probe qPCR Master Mix (NEB, M3004L) according to manufacturer’s protocol. *GAPDH* was used as control for gene expression. For assaying the relative expression of each gene, the 2^–ΔΔCt^ was determined using an ABI 7500 instrument (Applied Biosystems).

#### List of Primers

TaqMAN: *ZEB1* (Thermo Fisher Scientific, Hs00232783_m1), *ZEB2* (Thermo Fisher Scientific, Hs00207691_m1), *SNAI1* (Thermo Fisher Scientific, Hs00195591_m1), *SNAI2* (Thermo Fisher Scientific, Hs00950344_m1), *TWIST1* (Thermo Fisher Scientific, Hs01675818_s1), *GAPDH* (Thermo Fisher Scientific, 4326317E).

SYBR Green: *KRT14* (IDT, Hs.PT.58.4592110), *KRT19* (IDT,Hs.PT.58.4188708), *MEG3* ex 10-11 (IDT, Hs.PT.58.25190740), *GAPDH* (IDT, Hs.PT.39a.22214836), *KRT5* (IDT, Hs.PT.58.14446018), *TP63* (IDT, Hs.PT.58.2966111), *CDH3* (IDT, Hs.PT.58.39234242).

### Small RNAseq

The Microarray of small RNA data was published previously by our group by Hilmarsdottir and colleagues (Hilmarsdottir et al., 2015) and the small RNAseq data was published previously by Briem and colleagues (Briem et al., 2019a).

### miRNA qRT PCR

Total RNA was extracted with Tri-Reagent (Thermo Fisher Scientific, AM9738). The RNA was reverse transcribed using miRCURY LNA RT Kit (Qiagen, 339340) for cDNA synthesis reactions, according to manufacturer’s protocol. Quantitative RT-PCR analysis of miRNAs was performed using miRCURY LNA SYBR Green PCR Kit (Qiagen, 339346), according to manufacturer’s protocol. Gene expression levels were quantified using primers for: hsa-miR-127-3p (Qiagen, YP00204048), hsa-miR-409-3p (Qiagen, YP00204358), hsa-miR-411-5p (Qiagen, YP00204531), hsa-miR-493-3p (Qiagen, YP00204557). Normalization was done with U6 snRNA (Qiagen, YP00203907). The 2^–ΔΔCt^ was used determined using ABI 7500 instrument (Applied Biosystems) to calculate the relative expression of each gene.

### Allele Specific Expression Analysis

Total RNA was extracted with Tri-Reagent (Thermo Fisher Scientific, AM9738) and reverse transcription done using 1 μg of DNase I-treated total RNA using random hexamers (Thermo Fisher Scientific, N8080127) and SuperScript II Reverse Transcriptase (Thermo Fisher Scientific, 18064022) according to the manufacturer’s instructions. PCR primers were designed using Primer3 and Pyrosequencing primers were designed using PyroMark Assay Design 2.0 (Qiagen). The reverse PCR primer had a 5′-biotin modification and was HPLC-purified. Primers were synthesized by IDT 5′-TGGCCTTTTCTTCTCCTGAA, 5′-/5Biosg/TGACACATGGAAAGCACCAT and sequencing primer 5′-TCCGGGGTTACTGCCCT-3′. Polymerase chain reactions were performed in 50 μl using 10 ng of diluted cDNA or 10 ng of DNA, 1 U DreamTaq DNA polymerase (Fermentas, EP0701), 1X PCR buffer, 200 μM of dNTPs and 0,5 μM of each PCR primer. The following PCR protocol was used: 94°C for 2 min, followed by 50 cycles of 94°C for 1 min, 60°C for 1 min, 72°C for 1 min and 72°C for 9 min. To check the quality of the amplification, PCR products were analyzed by gel electrophoresis. Pyrosequencing were sequenced using the PyroMark Q24 system (Qiagen), following the manufacturer’s instructions. For the ASE SNP, DNA and RNA (cDNA) were pyrosequenced simultaneously. The proportions of individual alleles for the SNP were obtained using the PyroMark Q24 software version 1.0.10 (Qiagen). Genomic DNA from D492M was examined to confirm the heterozygosity.

### Clinical Cohort

RNA from breast cancer patients (diagnosed in the years 1987–2003) and relevant patient data was obtained from the Department of Pathology Landspitali – The National University Hospital of Iceland. Informed consent was obtained from patients involved in this study according to the national guidelines. The study was approved by The Icelandic Data Protection Commission (2001/523 and 2002/463) as well as the National Bioethics Committee of Iceland (VSN-11-105-V2). 119 samples were used in the study assigned to the following subgroup: 33 luminal A, 24 luminal B, 22 Basal, 12 ErbB2, 10 Normal and 18 not classified. cDNA was synthesized from 2 μg of total RNA using Random Hexamers primers (Thermo Fisher Scientific, N8080127) and RevertAid First Strand cDNA Synthesis Kit (Thermo Fisher). *MEG3* mRNA expression level was measured with the previously described qRT-PCR primers and TBP (Applied Biosystems, 4326322E) was used as a reference gene.

### Western Blot Assay

Cells were washed with cold Phosphate Buffered Saline (PBS) and lysed in radio immunoprecipitation assay (RIPA) buffer with phosphatase and protease inhibitors (Halt Protease Inhibitor Cocktail, Thermo Fisher Scientific, 78430) for 10 min on ice and scraped with cell scraper. Protein concentration was measured using Bradford reagent (BioRad, 5000002). Equal amounts of protein (5–15 μg) were separated on 10% NuPage Bis-Tris gels (Invitrogen, NP0301PK2) with NuPage MES running buffer (Thermo Fisher Scientific, NP0002) and transferred with NuPage Transfer buffer (Thermo Fisher Scientific, NP0006-1) to polyvinylidene fluoride (PVDF) membranes Millipore Imobilion-FL transfer membrane, pore size 0,45 μM (Millipore, IPFL00010). The membranes were blocked with Odyssey Blocking buffer (TBS) (LiCor, 927-500) and incubated with primary antibodies overnight at 4°C. List of antibodies: keratin 14 (KRT14; Abcam, Ab15461), keratin 19 (KRT19; Abcam, Ab7754), P-cadherin (CDH3; Cell signaling, CS2130), tumor protein p63 (TP63, Abcam, Ab124762), keratin 5/6 (KRT5/6; Invitrogen, 180267), Actin (Licor, 926-42212). Actin was used as loading control. Secondary antibodies were mouse or rabbit IRDey (Li-Cor 926-32213, 926-32212, respectively) used at 1:10.000 for 1 h at room temperature (RT) and detected and quantified using the Odyssey Infrared Imaging System (Li-Cor Fluorescent signal was detected by Odyssey image system (Li-Cor) and converted to gray scale.

### Cell Migration Assay

Cell migration was examined by using trans-well Boyden chambers with 8 μm pore size (Corning, 353097). Briefly, 3 × 10^3^ cells were resuspended in 250 μl H14 medium and seeded on the trans-well inserts in 24-well plate (Corning, 353047). H14 media with 10% FBS was added to the lower chamber, below filter. Cells were incubated for 48 h in 5% CO_2_ at 37°C. Non-migratory cells from the upper part of the filter were removed with cotton swab and washed 3 times with 1× PBS. The filters were then fixed with methanol and stained with DAPI (Sigma, D9542-1MG). Cells were photographed in three random fields EVOS FL Auto 2 imaging system (ThermoFisher). Pictures were analyzed with ImageJ Software.

### Low Attachment Assay

Anchorage independent growth was examined using 24-well ultra-low attachment plates (Corning, 3473). Briefly, D492 and D492M cells were single cell filtered and 500 cells/well were seeded into EGM5 media and cultured for 9 days. The growth of colonies was quantified under the microscope, counting all the colonies bigger than 40 μm.

### Apoptosis Assay

Resistance to chemically induced apoptosis was examined by inducing the cells with 10 μM camptothecin [CPT, Sigma-Aldrich, C9911)] in 96-well plate format (Corning, 353072). and quantified using IncuCyte Caspase-3/7 Reagents (Essen Bioscience, 4440) on IncuCyte Zoom (Essen Bioscience) according to the manufacturer’s instructions.

### Lentivirus Packaging and Transfection

The packaging of lentiviral expression constructs into pseudoviral particles, was performed with the psPAX2 (Addgene, 12260) and PMDG.2 (Addgene, 12259) plasmids using Turbofect (Thermo Fisher Scientific, R05319) in HEK-293T cells. The supernatant was harvested after 48 and 72 h and filtered through 0,45 μm pore filter. For infection, cells were plated on T25 flasks, so they were 70–80% confluent following day and were infected with 1 ml of viral particles and 1 ml of fresh media in the presence of 8 μg/ml polybrene. Lentivirus-transduced cells were selected with antibiotics or sorted by FACS (Sony SH800), based on fluorescent dye to obtain stable pool of clones. The altered expression of *MEG3* was determined by qRT-PCR.

The list of lentiviral expression constructs (plasmids) used in the study and their selection marker (with final concentration in case of antibiotics): pLenti_sgRNA(MS2)_zeo (Zeocin Invitrogen 4 μl/ml), pLenti_dCas9-VP64_Blast (Blasticidin, 2 μg/ml), pLenti_dCas9-KRAB_mCherry (mCherry fluorescence), SAM MS2-P65-HSF1 Plasmids (Hygromycin 1 μl/ml).

### CRISPRi/CRISPRa

To perform CRISPRi and CRISPRa, two vectors were used. First, vector with dCAS9 with effector domain KRAB (pLenti_dCas9-KRAB_mCherry, Genscript) and VP64 (pLenti_dCas9-VP64_Blast, Genscript) effector domain for CRISPRi and CRISPRa, respectively, was incorporated, using lentiviral transfection. Subsequently, vector with designed gRNA targeting specific site of our gene of interest *MEG3* was incorporated, in second round of lentiviral transfection. In case of gain of function studies with CRISPRa, one additional helper plasmid SAM (SAM MS2-P65-HSF1 Plasmids, Genscript) was used to further increase activation.

The sequence of gRNA for overexpression of *MEG3*: Guide 1: GCTCTCCGCCGTCTGCGCTA, the sequence of gRNA for downregulation of *MEG3*: Guide 2: GCGGGTGAGGGATCCTCTCGT, the sequence of gRNA for negative control: GCTTAGTTACGCGTGGACGA were cloned into pLenti_sgRNA(MS2)_zeo (Genscript).

### Statistical Analysis

**FIGURE 1:**
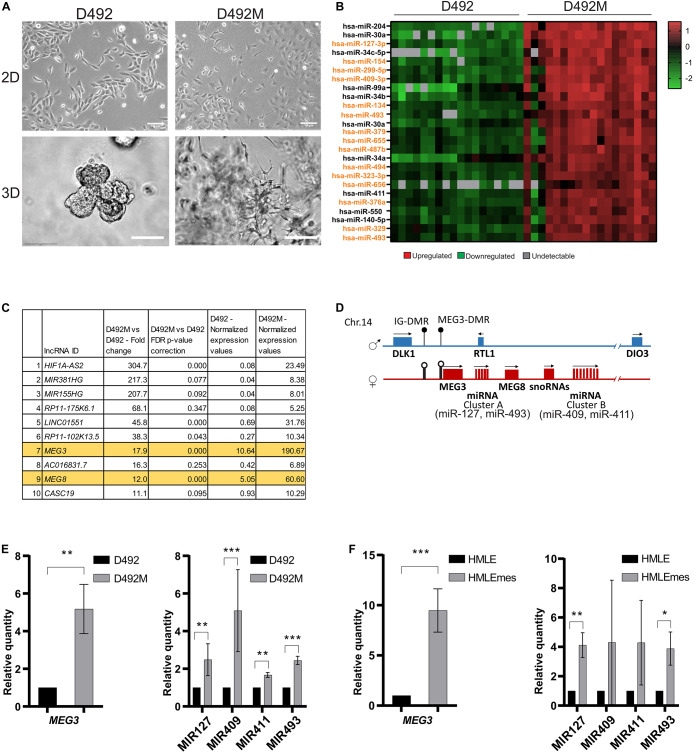
The DLK1-DIO3 locus is upregulated in breast epithelial stem cells undergoing EMT. **(A)** D492 and D492M generate branching and mesenchymal structures in 3D culture, respectively. In 2D culture, D492 is cuboidal in shape and D492M is more spindle shaped. Scale bar = 100 μm. **(B)** Majority of the top upregulated miRNAs in D492M are from the DLK1-DIO3 locus. Microarray heat map showing top 25 upregulated miRNAs in D492M compared to D492. 15 of them are from the DLK1-DIO3 locus (highlighted in orange). **(C)** LncRNAs from the DLK1-DIO3 locus are among the most upregulated in D492M. RNAseq data showing top ten differentially expressed lncRNA, with lncRNAs from DLK1-DIO3 locus (*MEG3* and *MEG8*) highlighted in orange. **(D)** Schematic figure of the DLK1-DIO3 locus. The DLK1-DIO3 locus is located on chromosome 14 and is imprinted. It contains three paternally expressed protein coding genes (*DLK1*, *RTL1*, and *DIO3*) and many maternally expressed non-coding genes, among them lncRNAs (*MEG3* and *MEG8*) and over 50 miRNAs, among them MIR127 and MIR493 located in cluster A and MIR409 and MIR411 located in cluster B and numerous C/D-box-containing small nucleolar RNAs (snoRNAs). DMR – differentially methylated region, filled circles represent methylated DMRs, and unfilled represent unmethylated DMRs. **(E)** Upregulation of selected ncRNA from the DLK1-DIO3 locus verified with qRT-PCR. Graphs showing higher expression of *MEG3* in D492M compared to D492 (left) and higher expression of four representative miRNAs (MIR127, MIR493, MIR409, and MIR411) at the DLK1-DIO3 locus in D492M compared to D492 (right). Results shown as mean ± SD. Unpaired *t*-test was used to test significance: **p ≤ 0.01; ***p ≤ 0.001; *n* = 3. **(F)** ncRNAs from the DLK1-DIO3 locus are upregulated in HMLEmes. qRT-PCR showing higher expression of *MEG3* and representative miRNAs (MIR127, MIR493, MIR409, and MIR411) from DLK1-DIO3 locus in HMLEmes compared to HMLE. Results shown as mean ± SD. Unpaired *t*-test was used to test significance: *p ≤ 0.05; **p ≤ 0.01; ***p ≤ 0.001; *n* = 3.

**FIGURE 2:**
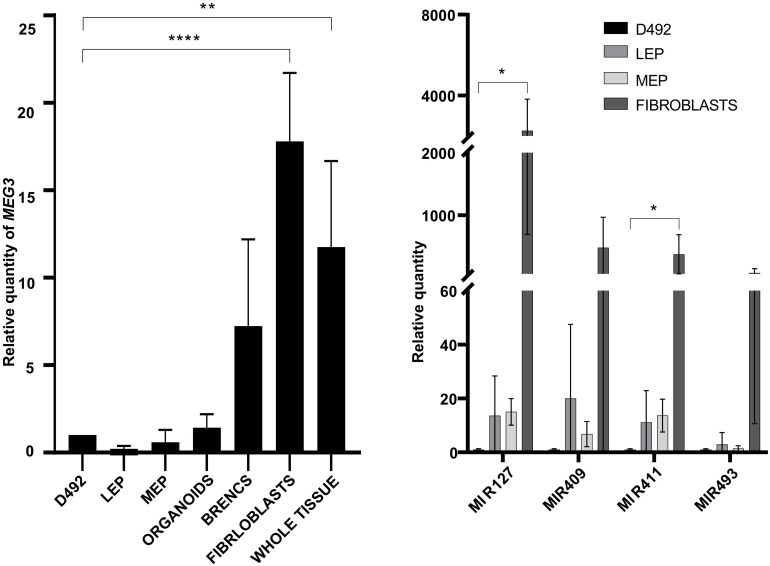
The ncRNA from the DLK1-DIO3 locus are highly expressed in stromal cells and whole tissue compared to epithelial cells. qRT-PCR showing *MEG3* expression is higher in breast stromal cells (fibroblasts) and whole tissue than in D492 (left). (LEP – luminal epithelial cells, MEP – myoepithelial cells, BRENCs – breast endothelial cells). Results shown as mean ± SD. One-way ANOVA with Dunnett’s multiple comparisons test was used to test significance: **p ≤ 0.01; ****p ≤ 0.0001; *n* = 3. Expression of representative miRNAs MIR127 and MIR411 at the DLK1-DIO3 locus is higher in breast fibroblast than in D492 (right). LEP – luminal epithelial cells, MEP - myoepithelial cells. Results shown as mean ± SD. One-way ANOVA with Dunnett’s multiple comparisons test was used to test significance: *p ≤ 0.5; *n* = 3.

Statistical differences of qRT-PCRs (Figures 1E,F, Figures 5A,B, and Figures 7A–C) and functional assay (Figures 8B–E) between samples were assessed with unpaired Student *t*-test. Statistical differences in Figure 8A was calculated using multiple unpaired Student *t*-test per row. Statistical differences of quantifications of western blots (Figures 7B,C) among samples were assessed using one-way ordinary ANOVA, followed by Tukey’s multiple comparison test. Statistical differences in Figure 4A (left) was calculated using Kruskal Wallis Test (one-way ANOVA on ranks). Statistical analysis of qRT-PCRs in Figure 2 were assessed with One-way ANOVA with Dunnett’s multiple comparisons test. All statistical analyses were performed in GraphPad Prism. *P*-values below 0,05 were considered significant (^∗^*p* ≤ 0.05; ^∗∗^*p* ≤ 0.01; ^∗∗∗^*p* ≤ 0.001; ^****^p ≤ 0.0001).

## Results

### *MEG3* Is Highly Expressed in Cell Lines With a Mesenchymal Phenotype and in the Stromal Compartment of Breast Tissue

D492 and D492M are isogenic cell lines with stem cell and mesenchymal properties, respectively. D492 cells acquire cuboidal shape in 2D culture, and form branching structures in 3D culture, akin to terminal duct lobular units (TDLU) in the breast. In contrast, D492M is elongated and spindle-shaped in 2D culture and in 3D culture it forms irregular mesenchymal-like colonies (Figure 1A). We have previously shown that MIR203a and the MIR200 family are downregulated in D492M and their expression is essential for the epithelial phenotype (Hilmarsdottir et al., 2015; Briem et al., 2019a). Of miRNAs upregulated in D492M, the miRNAs at the DLK1-DIO3 locus are prominent. A microarray analysis of miRNA expression demonstrated that 15 of the 25 most highly expressed miRNAs in D492M compared to D492 belong to the DLK1-DIO3 locus (Figure 1B). Furthermore, small RNA sequencing revealed that 33 of the miRNAs belonging to the DLK1-DIO3 miRNA cluster have more than 1,5-fold increased expression in D492M compared to D492 (Supplementary Figure 1). Moreover, total RNA sequencing of D492 and D492M, revealed that *MEG3* and *MEG8* are amongst the most upregulated lncRNAs in D492M (Figure 1C). The non-coding part of the DLK1-DIO3 locus consists of maternally expressed lncRNAs *MEG3* and *MEG8* and miRNAs grouped into two clusters (Figure 1D). To confirm the sequencing results, we selected four representative miRNAs from the DLK1-DIO3 locus, two from each cluster (MIR127 and MIR493 from cluster A, MIR409 and MIR411 from cluster B). These miRNAs as well as the lncRNA *MEG3* had higher expression, as revealed by qRT-PCR, in D492M compared to D492 (Figure 1E). In another isogenic EMT cell model, HMLE (epithelial) and HMLEmes (mesenchymal variant) both *MEG3* and the representative miRNAs were more highly expressed in HMLEmes compared to HMLE (Figure 1F). Thus, our data suggests that increased *MEG3* expression is not a stochastic event but consistently associates with EMT induction in breast epithelial cell lines.

Next, we analyzed the expression of *MEG3* and miRNAs from the DLK1-DIO3 locus in primary cells from three healthy donors. We found that the expression of *MEG3* is higher in purified stromal cells (fibroblasts) than in epithelial cells (D492, luminal epithelial cells, myoepithelial cells and organoids; Figure 2, left). Interestingly, expression of *MEG3* in whole breast tissue lysates is closer to fibroblast expression levels than epithelial cells (Figure 2, left). This finding is most likely explained by the richness of stroma in normal breast tissue, whereas organoids contain only the epithelial cells. A similar pattern is seen with the four representative miRNAs, where MIR127 and MIR411 have higher expression in fibroblasts compared to their expression in D492 (Figure 2, right).

We next acquired a list of genes correlated the expression of MEG3 using the GOBO (Gene expression-based Outcome for Breast Cancer Online) dataset and submitted the list to DAVID (the database for annotation, visualization and integrated discovery, version 6.7) (Huang et al., 2009a, b) to identify pathways associated with *MEG3*. Herein, the expression of *MEG3* correlates with expression of extracellular matrix genes, which are in line with the observations of a high expression of *MEG3* in cells found in the stromal compartment (Supplementary Figure 2A). Using analysis of publicly available NGS data using MiPanda (Niknafs et al., 2018) we found positive correlation of *MEG3* with common EMT markers in normal breast and breast cancer (Supplementary Figure 2B). Many of these have a correlation coefficient > 0.3 (Spearman correlation) which is considered a fair positive correlation (Chan, 2003). Interestingly, even more genes are positively correlated to *MEG3* expression in breast cancer as compared to normal breast tissue (Supplementary Figure 2B).

Collectively, the lncRNA *MEG3* and miRNAs from DLK1-DIO3 locus are highly expressed in the mesenchymal compartment compared to epithelial breast tissue and their expression positively correlate with numerous mesenchymal genes and EMT markers.

### MEG3 Is Imprinted in Both D492 and D492M

**FIGURE 3:**
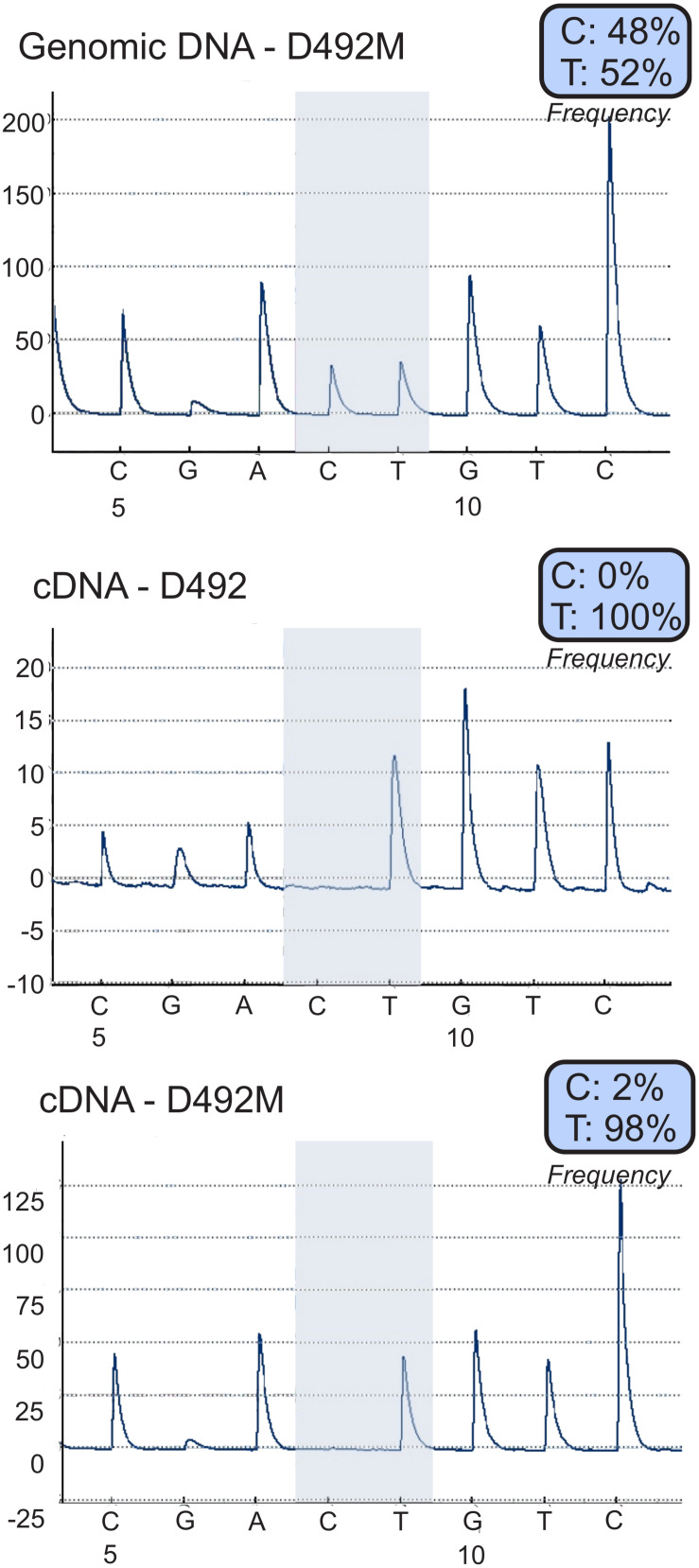
Increased expression of *MEG3* in D492M is not caused by loss of imprinting. Allele specific expression analysis using pyro-sequencing shows that the increased expression of *MEG3* in D492M is not caused by loss of imprinting, but increased expression of the already expressed allele, the expression remains mono-allelic. Analyzed sequence: GAGCAC/TGTCCCA.

The DLK1-DIO3 locus is imprinted and regulated by DNA methylation (Cui et al., 2018). Using pyrosequencing (Harrington et al., 2013) covering a heterozygous SNP (C/T) in *MEG3* (rs4906024) we confirmed monoallelic expression of *MEG3* in both D492 and D492M, with expression in both cell lines being from the T allele (Figure 3). As both cell lines are diploid at the *MEG3* locus on a DNA level a C/T ratio of 50% is expected which is consistent with the 48% C-allele prominence observed. On the mRNA deviation from expected monoallelic expression was not detected as results showed zero C allele expression in D492 and 2% in D492M. Hence, increased expression of *MEG3* in D492M is not caused by loss of imprinting. The expression remains monoallelic confirming that the increased expression originates from the non-imprinted allele.

**FIGURE 4:**
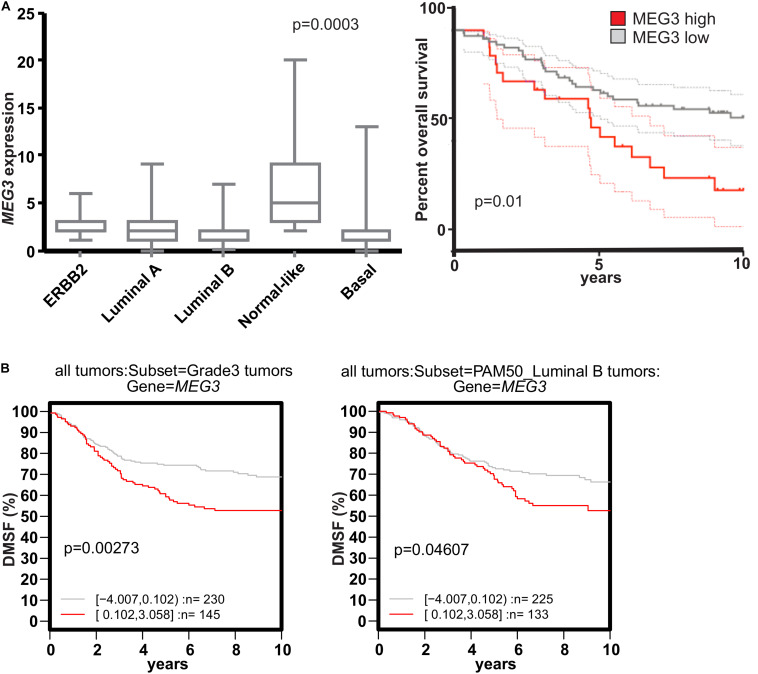
*MEG3* expression negatively correlates with breast cancer prognosis. **(A)** High *MEG3* expression decreases overall survival in breast tumors. qRT-PCR shows that *MEG3* expression (Numbers of tumors per group: ErBB2 = 12, Luminal A = 33, Luminal B = 24, Normal = 10, Basal = 22) is significantly higher in normal-like (NL) breast cancer (left; *p* = 0.0003). High MEG3 expression is correlated with low overall patient survival (NL tumors omitted). Kruskal Wallis Test (or one-way ANOVA on ranks) was used to test the significance (right; *p* = 0.01). **(B)** High *MEG3* expression decrease distant metastasis free survival in grade three and Luminal B type tumors. Kaplan-Meier plot showing data from the online GOBO database: high expression of *MEG3* decrease DMSF (distant metastasis free survival), of poorly differentiated (grade 3) tumors (left; *p* = 0.00373) and luminal B type tumor (right; *p* = 0.04607).

**FIGURE 5:**
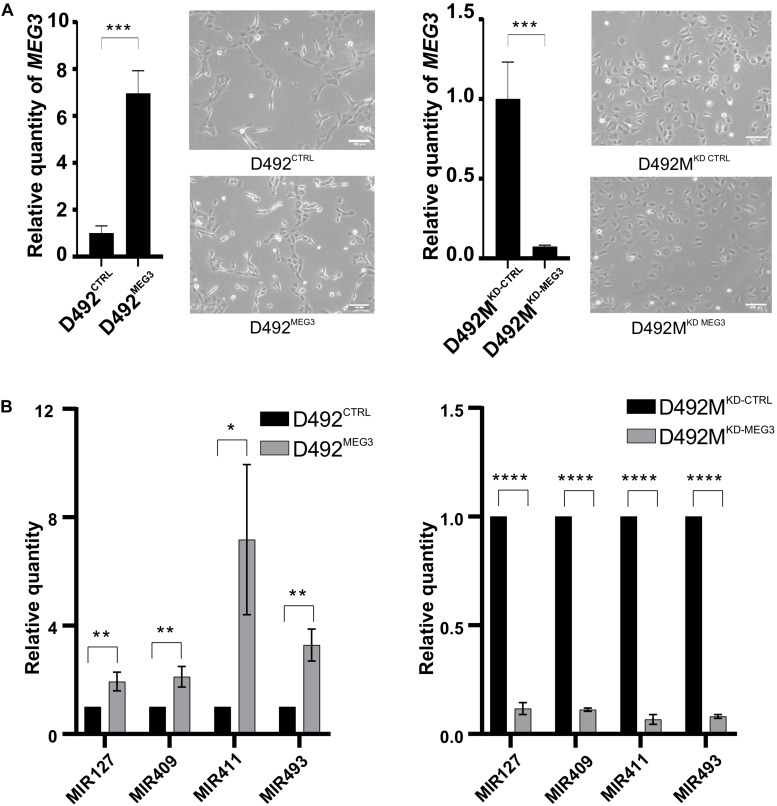
Concomitant expression of non-coding RNAs from DLK-DIO3 locus with *MEG3*. **(A)** Overexpression and knock-down of *MEG3* in D492 and D492M, respectively. qRT-PCR confirming upregulation of *MEG3* in D492 (D492^MEG3^) compared to D492 with scrambled control (D492^CTRL^; left). Phase contrast pictures of D492^CTRL^ and D492^MEG3^ (below). qRT-PCR confirming knock-down of *MEG3* in D492M (D492M^KD–MEG3^) compared to D492M with scrambled control (D492M^KD–CTRL^; right). Results shown as mean ± SD. Unpaired *t*-test was used to test significance: ***p ≤ 0.001; ****p ≤ 0.0001; *n* = 3. Phase contrast pictures of D492M^KD–CTRL^ and D492M^KD–MEG3^ (below). Scale bar = 100 μm. **(B)** miRNAs form the DLK1-DIO3 locus are upregulated with overexpression of *MEG3* and downregulated with knock-down of *MEG3*. qRT-PCR shows increased expression of four representative miRNAs from the DLK1-DIO3 locus in D492^MEG3^ compared to D492^CTRL^ (left) and their decreased expression in D492M^KD–MEG3^ compared to D492M^KD–CTRL^ (right). Results shown as mean ± SD. Unpaired *t*-test was used to test significance: *p ≤ 0.05 **p ≤ 0.01; ****p ≤ 0.0001; *n* = 3.

### Increased Expression of *MEG3* Is Negatively Correlated With Survival of Breast Cancer Patients

EMT has been suggested to promote metastatic behavior of epithelia-originating cancer (Felipe Lima et al., 2016) and, in addition, our data shows association of *MEG3* expression with the mesenchymal phenotype. We therefore investigated *MEG3* expression levels in different subtypes of breast cancer. We have evaluated the expression of *MEG3* in clinically well-defined breast tumors. Herein, normal like (NL) breast tumors had significantly higher expression of *MEG3* with a *p*-value of 0.0003 (Figure 4A, left). Survival analysis of all tumor samples showed reduced, but not significant overall survival in patients with high *MEG3* expression. However, as the normal-like tumors have in recent years been subjected to scrutiny as a possible misclassification due to low tumor cellularity and thus, high proportion of normal tissue. In light of our results showing high expression of *MEG3* in breast stromal tissue, and uncertainty that measured *MEG3* expression in the normal-like subgroup is representative of the primary tumor, we omitted NL breast tumors from the survival analysis (Elloumi et al., 2011; Prat and Perou, 2011; Yersal and Barutca, 2014). The results show significant worse overall survival of patients with high *MEG3* expression (Figure 4A, right). Corroborating our findings, using the GOBO database (Ringnér et al., 2011)^1^, we found that high *MEG3* expression reduces distant metastasis free survival (DMSF) of patients with poorly differentiated (grade 3) tumors (Figure 4A, left) and patients with luminal B tumors (Figure 4B, right).

### Increased Expression at the DLK1-DIO3 Locus Contributes to the Basal and Mesenchymal Phenotype

To explore the functional role of *MEG3* in D492 and D492M, we established sublines with altered expression of *MEG3*. Using the CRISPRa approach (Cheng et al., 2013), we generated a D492 cell line with stable overexpression of *MEG3* (D492^MEG3^). A control cell line was generated using a scrambled sgRNA (D492^CTRL^). Furthermore, we used the CRISPRi approach (Gilbert et al., 2013; Qi et al., 2013), to generate knockdown of *MEG3* in D492M (D492M^KD–MEG3^) and a control cell line was generated using scrambled sgRNA (D492M^KD–CTRL^). The increase of *MEG3* expression was about seven-fold in D492^MEG3^ compared to D492^CTRL^ as determined by qRT-PCR (Figure 5A, left). Downregulation of *MEG3* in D492M^KD–MEG3^ was more prominent, with about 20-fold reduced expression compared to D492M^KD–CTRL^ (Figure 5A, right). Having established stable overexpression and downregulation of *MEG3* in D492 and D492M, we re-evaluated the epithelial/mesenchymal phenotypes of D492 and D492M, respectively. Based on phase contrast images, no obvious difference in phenotype could be seen between D492^MEG3^ and D492^CTRL^ or D492M^KD–MEG3^ and D492M^KD–CTRL^ (Figure 5A, below). Interestingly, expression of the representative miRNAs located on the DLK1-DIO3 locus is increased in D492^MEG3^ compared to D492^CTRL^, to similar levels as seen in D492M (Figure 5B, left). Conversely, the expression of representative miRNAs is downregulated in D492M^KD–MEG3^ compared to D492M^KD–CTRL^ (Figure 5B, right). Thus, it appears, that the expression of miRNAs from the DLK1-DIO3 locus is concomitant with *MEG3* expression. To test, if that holds true, we used the cBioPortal and explored correlation of *MEG3* with miRNAs using data on invasive breast cancer from the Cancer Genome Atlas (TCGA) (Cancer Genome Atlas Network, 2012) we found that of 40 miRNAs that had positive correlation over 0,3 (Person score) with *MEG3*, 30 were located at the DLK1-DIO3 locus (with other miRNAs from the locus not being in the dataset; Table 1). This suggests that *MEG3* may be used as a marker for the expression of ncRNAs from the DLK1-DIO3 locus.

**TABLE 1:** MiRNAs from the DLK1-DIO3 locus positively correlate with *MEG3* expression.

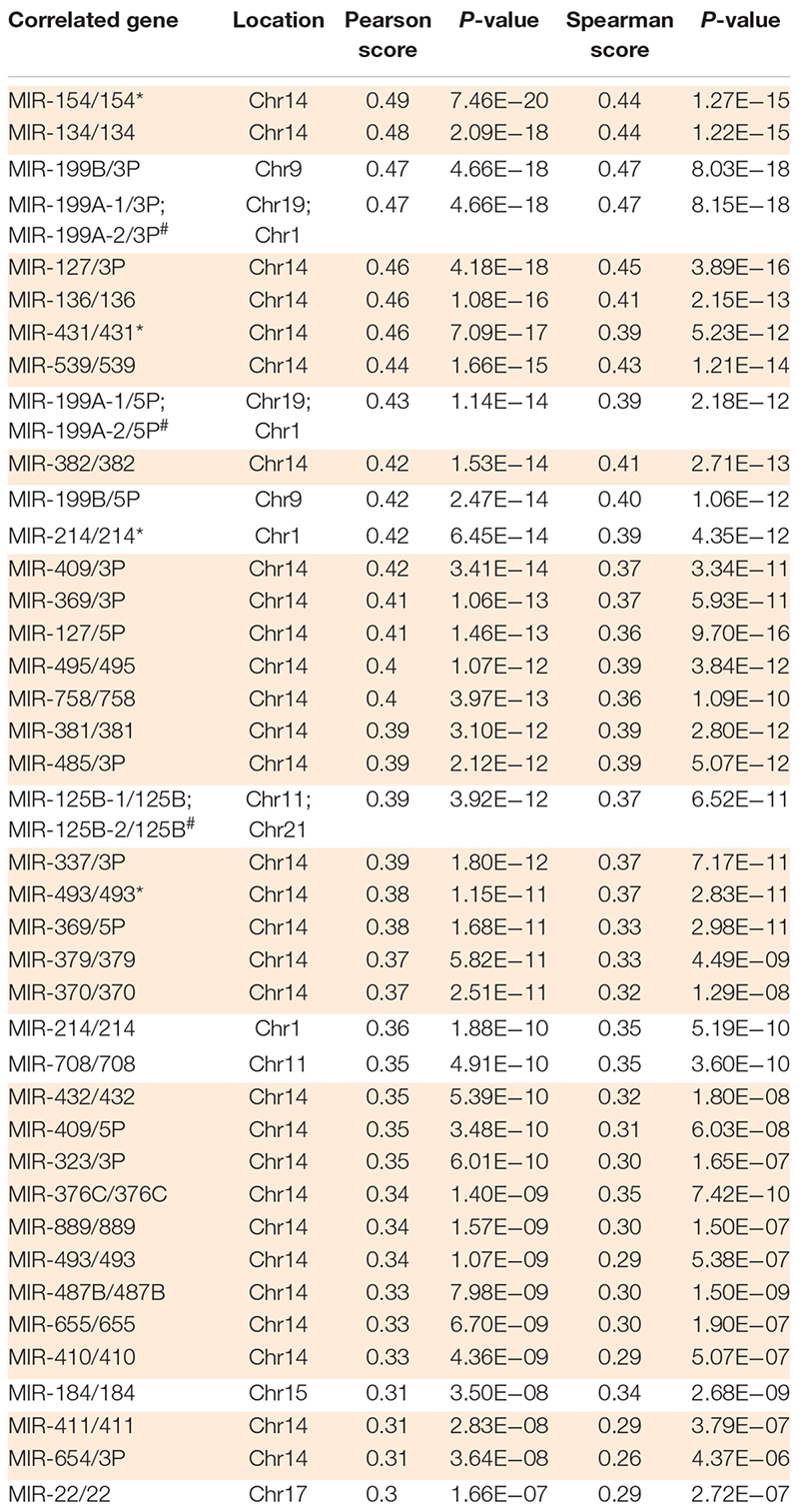

*Out of 40 miRNAs that positively correlate with *MEG3* in breast cancer (with correlation over 0.3), 30 are from the DLK1-DIO3 locus, highlighted in orange (TCGA, Nature 2012 data set). ^#^Due to sequence similarities, these two miRNAs are indistinguishable in the sequencing data used.*

**FIGURE 6:**
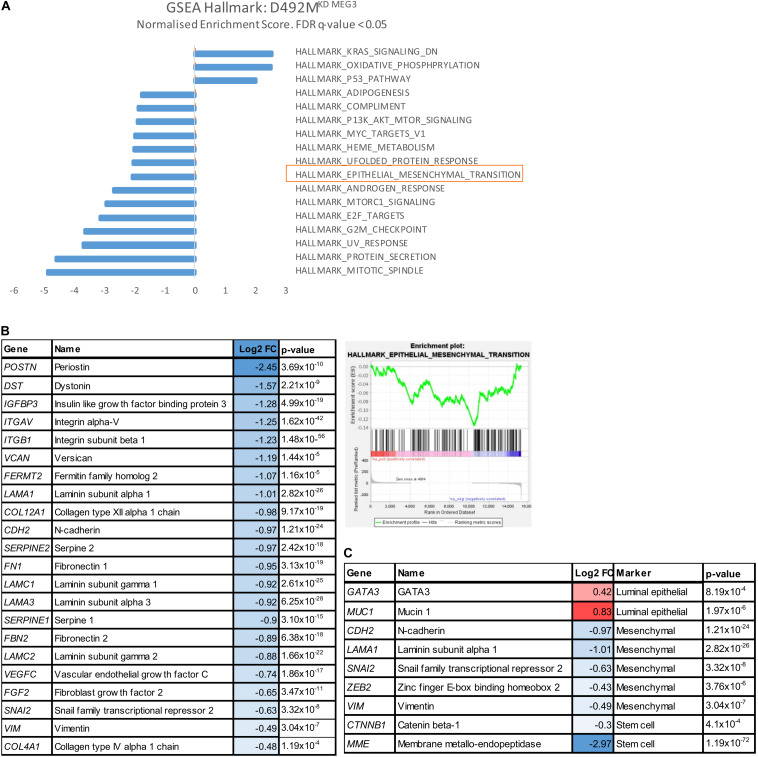
Knock down of *MEG3* in D492M decrease mesenchymal markers. **(A)** Epithelial-mesenchymal transition gene set is enriched pathway in D492M^KD–MEG3^ (highlighted in orange). Bar plot of Gene Set Enrichment Analysis (GSEA) with Hallmark dataset showing all significantly (False discovery rate-FDR *q* ≤ 0.05) enriched pathways in D492M^KD–MEG3^. Gene set Epithelial-mesenchymal transition has normalized enrichment score (NES) of –2.06 and FDR *q* = 0.014. **(B)** Knock-down of *MEG3* correlates with downregulation of mesenchymal genes relevant for breast cells. Enrichment plot showing the Enrichment Score (ES) of the genes in the Hallmark gene set Epithelial-mesenchymal transition in D492M^KD–MEG3^. The genes are overrepresented toward the top the ranked list of D492M^KD–MEG3^ (right). Table with relevant genes for breast cells, significantly (*p* ≤ 0.05) deregulated, from the Hallmark gene list epithelial-mesenchymal transition showing Log2 fold change (FC; left). **(C)** Luminal epithelial markers (*GATA3* and *MUC1*) are upregulated, while myoepithelial (*KRT14*), mesenchymal (*CDH2*, *LAMA1*, *SNAI2*, *VIM*, and *ZEB2*) and stem cell (*CTNNB1* and *MME*) are downregulated with knock down of *MEG3*. Genes from literature-based list of markers significantly (*p* ≤ 0.05) differentially expressed in D492M^KD–MEG3^ vs D492M^KD–CTRL^.

Next, we conducted RNA sequencing of our cell lines with stably altered expression levels of *MEG3* focusing on the analysis of D492M^KD–CTRL^ vs D492M^KD–MEG3^. There were 1235 significantly differentially expressed genes, with symmetric distribution over genes downregulated and upregulated in D492M^KD–MEG3^, shown in the volcano plot (*p* < 0.05; Supplementary Figure 3A), with the list of top 30 up and down-regulated genes in D492M^KD–MEG3^ (Supplementary Figure 3B). To identify unifying biological them from RNA-sequencing data, we performed Gene Set enrichment analysis (GSEA). These gene sets consist of the defined gene lists, based on biological knowledge about biochemical pathways and co-expression data. Using the Hallmark dataset, one of the significantly, downregulated set of genes in D492M^KD–MEG3^ was the epithelial-mesenchymal transition gene set, with normalized enrichment score (NES) of −2.03 and False discovery rate (FDR) *q* = 0.023 (Figure 6A). These genes define epithelial-mesenchymal transition, as in wound healing, fibrosis and metastasis. The genes belonging to this gene set are overrepresented toward the top of the ranked list, based on fold change of D492M^KD–CTRL^ vs D492M^KD–MEG3^ (Figure 6B, right). A manually curated list of mesenchymal genes from the Hallmark EMT dataset that are downregulated in D492M^KD–MEG3^ is shown in Figure 6B, left. Further analysis of the RNA sequencing data of D492M^KD–MEG3^ vs D492M^KD–CTRL^, using common literature-based markers of breast tissue has showed that luminal epithelial markers *GATA3* and *MUC1* are upregulated, while myoepithelial *KRT14*, mesenchymal *VIM*, *ZEB2*, *SNAI2*, *LAMA1*, *CDH2*, and stem cell *MME*, *CTNNB1* are downregulated with knock down of *MEG3* (Figure 6C).

**FIGURE 7:**
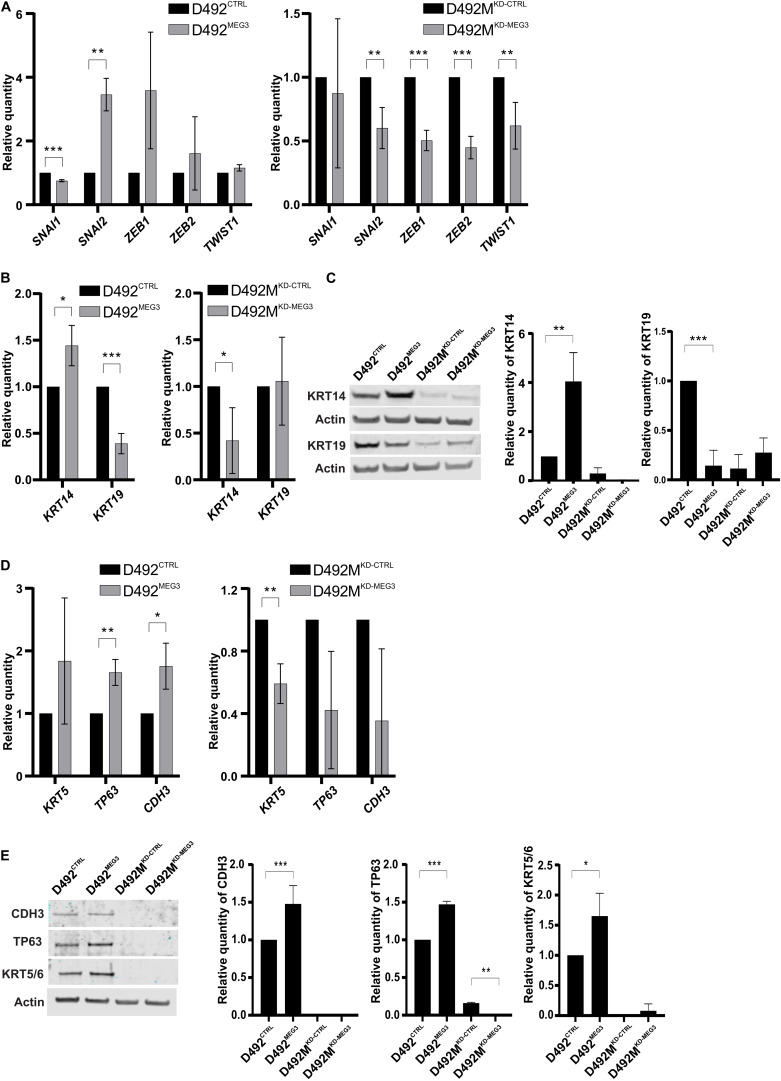
*MEG3* induce partial EMT. **(A)** *MEG3* increases expression of EMT transcription factors in D492 and the knock down of *MEG3* decrease expression of TF-EMT in D492M. qRT-PCR showing D492^MEG3^ increased expression of transcription factors (TF) *SNAI2* compared to D492^CTRL^ (left) and decreased expression of EMT related TF *SNAI2*, *ZEB1*, *ZEB2* and *TWIST1* in D492M^KD–MEG3^ compared to D492M^KD–CTRL^ (right). Results shown as mean ± SD. Unpaired *t*-test was used to test significance: **p ≤ 0.01; ***p ≤ 0.001; *n* = 3. **(B)** *MEG3* increases expression of myoepithelial marker *KRT14* and decrease expression of luminal epithelial marker *KRT19* on mRNA level. qRT-PCR showing D492^MEG3^ has increased expression of *KRT14* and decrease expression of *KRT19* compared to D492^CTRL^ (left). D492M^KD–MEG3^ has decreased expression of *KRT14* compared to D492M^KD–CTRL^ (right). qRT-PCR results shown as mean ± SD. Unpaired *t*-test was used to test significance: *p ≤ 0.05; ***p ≤ 0.001; *n* = 3. **(C)** qRT-PCR results confirmed on protein level. Representative pictures of western blot (WB) with its quantification (below). D492^MEG3^ has increased protein level of KRT14 and decreased protein level of KRT19 compared to D492^CTRL^. WB results shown as mean ± SD. One-way ordinary ANOVA, followed by Tukey’s multiple comparison test was used to test significance: **p ≤ 0.01; ***p ≤ 0.001; *n* = 3. **(D)** *MEG3* increase expression of myoepithelial markers *TP63* and *CDH3* and knock-down of *MEG3* decrease expression of myoepithelial marker *KRT5*, on mRNA level. qRT-PCR showing D492^MEG3^ has increased expression of *TP63* and *CDH3* compared to D492^CTRL^ (left). D492M^KD–MEG3^ has decreased expression of *KRT5* compared to D492M^KD–CTRL^ (right). qRT-PCR results shown as mean ± SD. Unpaired *t*-test was used to test significance: *p ≤ 0.05; **p ≤ 0.01; *n* = 3. **(E)** qRT-PCR results confirmed on protein level. Representative pictures of western blot (WB) with its quantification (below). D492^MEG3^ has increased protein level of CDH3 (P-cad), TP63 (p63) and KRT5 compared to D492^CTRL^. D492M^KD–MEG3^ has decreased protein level of TP63 compared to D492M^KD–CTRL^. WB results shown as mean ± SD. One-way ordinary ANOVA, followed by Tukey’s multiple comparison test was used to test significance: *p ≤ 0.05; **p ≤ 0.01; ***p ≤ 0.001; *n* = 3.

**FIGURE 8:**
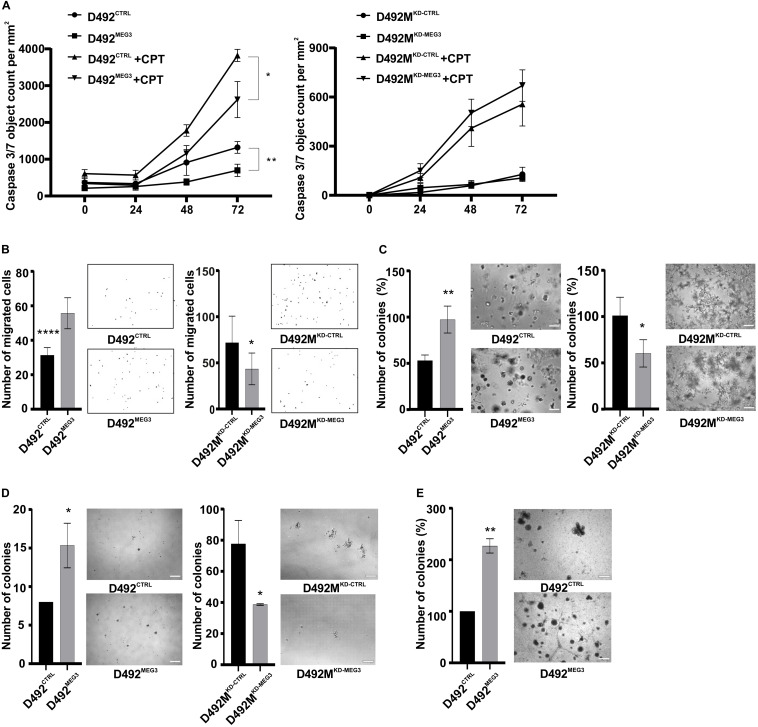
*MEG3* increases stem cell properties. **(A)** *MEG3* increase resistance to chemically induced apoptosis. Apoptosis assay: D492^MEG3^ is more resistant to chemically induced apoptosis compared to D492^CTRL^ (left). Data is analyzed on Incucyte Zoom and displayed as Caspase 3/7 object count/mm^2^. Results are shown as mean ± SD. Multiple unpaired Student *t*-test per row was used to test significance at 72-h time-point: *p ≤ 0.05; **p ≤ 0.01; *n* = 6. **(B)** *MEG3* increases migration and knock down of *MEG3* decreases migration through trans-well filters. Migration assay: D492^MEG3^ has increased migration rate compared to D492^CTRL^ (left), with representative pictures on side. D492M^KD–MEG3^ migrates less compared to D492M^KD–CTRL^ (right), with representative pictures on side. Quantification of number of migratory cells, analyzed using ImageJ software, results shown as mean ± SD. Unpaired *t*-test was used to test significance: *p ≤ 0.05; ****p ≤ 0.0001; *n* = 6. **(C)** *MEG3* increases clonogenic capacity in 3D culture in rBM (reconstitute basement membrane). Mammosphere assay: D492^MEG3^ has higher clonogenic capacity compared to D492^CTRL^ (left). D492M^KD–MEG3^ has lower clonogenic capacity compared to D492M^KD–CTRL^ (right). Data shown as % mean ± SD, *p ≤ 0.05; **p ≤ 0.01; *n* = 3. Scale bar = 500 μm. **(D)** *MEG3* increases clonogenic capacity in low attachment assay. D492^MEG3^ increases the formation of colonies compared to D492^CTRL^ (left), with representative pictures on side. D492M^KD–MEG3^ decreases the formation of colonies compared to D492M^KD–CTRL^ (right), with representative pictures on side. Results shown as % of mean ± SD. Unpaired *t*-test was used to test significance: *p ≤ 0.05; **p ≤ 0.01; *n* = 3. Scale bars = 200 μm. **(D)** Co-culture of D492^MEG3^ with endothelial cells (HUVECs) increases number and size of colonies and forms less branching compared to D492^CTRL^. Results shown as % of mean ± SD. Unpaired *t*-test was used to test significance: **p ≤ 0.01; *n* = 3. Representative pictures scale bars = 200 μm.

Expression of mesenchymal and basal markers was additionally confirmed on RNA level by qRT-PCR and on protein level western blot. Most of the core EMT-related transcription factors (EMT-TF) were affected by *MEG3*. D492^MEG3^ has increased expression of *SNAI2* compared to D492^CTRL^ (Figure 7A, left). On the other hand, D492M^KD–MEG3^ has decreased expression of *SNAI2*, *ZEB1*, *ZEB2* and *TWIST1* compared to D492M^KD–CTRL^ (Figure 7A, right). Luminal cytokeratin 19 (*KRT19*) and basal/myoepithelial cytokeratin 14 (*KRT14*) are also affected by manipulation of *MEG3* expression levels. Thus, D492^MEG3^ shows increased *KRT14* and decreased *KRT19* expression compared to D492^CTRL^ on both mRNA (Figure 7B, left) and protein level (Figure 7C, left). D492M^KD–MEG3^ shows decreased *KRT14* expression compared to D492M^KD–CTRL^ (Figure 7B, right). Furthermore, D492^MEG3^ shows increased expression of other myoepithelial markers such as *CDH3* (P-cad), *TP63* or *KRT5* compared to D492^CTRL^ as determined both at mRNA (Figure 7D, left) and protein level (Figure 7E). Also, D492M^KD–MEG3^ shows decreased expression of myoepithelial markers *KRT5* on mRNA level (Figure 7D, right) and of TP63 on protein level (Figure 7E, middle) compared to D492M^KD–CTRL^. This suggests that *MEG3* expression induces a shift toward a basal/myoepithelial phenotype. However, our cell lines with stably altered expression of *MEG3* do not show a significant switch in E-cadherin (CDH1) to N-cadherin (CDH2) expression (Supplementary Figure 4), which may explain why there are no clear changes in morphology.

### *MEG3* Induces Mesenchymal Properties and Stemness

As *MEG3* has previously been ascribed to have a role in pluripotency and stemness (Stadtfeld et al., 2010b; Kaneko et al., 2014), we asked how *MEG3* manipulation affects mesenchymal and stem cell properties of D492 and D492M. The expression of both aldehyde dehydrogenase (*ALDH1A3*) and integrin alpha 6 (*ITGA6*; Supplementary Figure 6), markers of stemness, is increased in D492^MEG3^ compared to D492^CTRL^. Next, we employed several functional assays to assess the effect of *MEG3* levels in D492 and D492M on mesenchymal and stem cell properties. D492^MEG3^ is more resistant to chemically induced apoptosis than D492^CTRL^ (Figure 8A). Migration can be assessed *in vitro* using the wound healing assay or by trans-well migration where the cells migrate toward a chemo-attractant. In the wound healing assay, D492^MEG3^ has slightly increased migration rate compared to D492^CTRL^, while D492M^KD–MEG3^ has decreased migration rate compared to D492M^KD–CTRL^ (Supplementary Figure 5A). In the trans-well migration assay, D492^MEG3^ has about two-fold increased migration rate compared to D492^CTRL^ and D492M^KD–MEG3^ has reduced migration rate compared to D492M^KD–CTRL^ (Figure 8B). *MEG3* manipulation, however, did not affect invasion in a transwell invasion assay (Supplementary Figure 5B). We performed mammosphere assays in rBM (reconstituted basement membrane, Matrigel) (Figure 8C) and in low attachment plates (Figure 8D), with comparable results. D492^MEG3^ increases the formation of colonies compared to D492^CTRL^ while D492M^KD–MEG3^ decreases the formation of colonies compared to D492M^KD–CTRL^. In addition, we co-cultured D492^MEG3^ with endothelial cells (HUVECs) and observed increased size of colonies and less branching compared to D492^CTRL^ (Figure 8E). Finally, manipulation of *MEG3* levels slightly affected proliferation rate of D492M^KD–MEG3^ compared to D492M^KD–CTRL^ (Supplementary Figure 5C).

## Discussion

In this study, we show that ncRNAs from the DLK1-DIO3 locus are highly expressed in stromal/mesenchymal cells in the breast and positively correlate with the expression of EMT genes in breast tissue. *MEG3* expression was monoallelic in both D492 and D492M and gain and loss of function studies have shown concomitant expression of *MEG3* with miRNAs from the DLK1-DIO3 locus, indicating that *MEG3* could be used as a marker for the expression of the non-coding RNAs from the locus. *MEG3* expression was shown to be negatively correlated with survival of breast cancer patients, particularly with the luminal B subtype. Furthermore, we demonstrate that enhanced *MEG3* expression accompanied by increased expression of the ncRNAs at the DLK1-DIO3 locus, contributes to partial EMT more correctly referred to as epithelial plasticity, seen by increased expression of EMT related TFs, increase of basal/mesenchymal markers and enhanced properties such as migration, resistance to apoptosis and clonogenic capacity.

We used an isogenic breast cell line model to study the expression pattern and functional role of ncRNAs, both miRNAs and lncRNAs, in EMT. Of interest was the largest miRNA locus in the human genome and the lncRNA *MEG3*, both within the DLK1-DIO3 imprinted region on chromosome 14. The non-coding part of the DLK1-DIO3 locus has higher expression in cells with mesenchymal phenotype (D492M) compared to cells with epithelial phenotype (D492). These results were validated in primary breast tissue and in another cellular model of EMT. Furthermore, we have shown that *MEG3* expression correlates with expression of extracellular matrix proteins, which are secreted by cells with a mesenchymal phenotype, and with mesenchymal genes in breast tissue. Data from pyrosequencing demonstrate that the expression of *MEG3* is monoallelic in both D492 and D492M indicating that the increased expression of *MEG3* in D492M is not due to loss of imprinting. We have shown that *MEG3* negatively correlates with survival of luminal B breast cancer patients and patients with grade 3 breast cancer. This is in line with a recent study where high expression of *MEG3* was identified to be a negative prognostic marker for breast cancer (Yao et al., 2019).

Many studies suggest *MEG3* as a tumor suppressor, largely due to the observation that *MEG3* expression is lower in tumor tissue compared to normal tissue (Sheng et al., 2014; Sun et al., 2014, 2016; Yin et al., 2015; Chak et al., 2017; Molina-Pinelo et al., 2018). Our data demonstrates that *MEG3* expression levels are comparable in whole normal breast tissue and in stroma (fibroblasts), however, the expression of *MEG3* in epithelial cells is much lower. There was considerable variation of the *MEG3* expression in breast tissue samples that could be partially due to different proportions of subset of fibroblasts associated with ducts vs TDLUs. There are studies confirming existence of, for instance, two distinct functionally specialized lineages of lobular vs ductal fibroblast (Morsing et al., 2016) or myoepithelial cells (Fridriksdottir et al., 2017), which could be identified by specific marker expression. Importantly, relative proportions of stromal and epithelial compartment are different in normal and cancerous human breast tissue. Breast cancers arise in vast majority from epithelial cells, with TDLUs being the predominant site of breast tumor occurrence (Tabar et al., 2014). Therefore, it would be expected that expression of *MEG3* is higher in normal breast tissue, as it comprises relatively more stromal cells compared to breast cancer tissue. In line with this, expression of *MEG3* from whole breast tissue is distorted as proportions of stroma vs epithelia in normal/cancer tissue are different, resulting in misleading interpretations. Using RNA only from unsorted normal tissue will mainly represents expression of stromal cells. Therefore, it is crucial to use a proper control when comparing expression of genes in normal vs tumor tissue. Single-cell RNA-sequencing or sorted stromal and epithelial cells would give more informative results as it would enable distinctions between epithelial and stromal tissue compartments. In this paper we show that *MEG3* expression negatively correlates with survival in breast cancer, particularly in grade three tumors and the luminal B subtype. However, our study does not determine if the high *MEG3* expression represents increased stromal infiltration in the tumors or elevated expression in cancer cells.

Another reason for classifying *MEG3* as tumor suppressor is its action on stabilization of p53 (Ghafouri-Fard and Taheri, 2019). However, inactivation of p53 is a frequent event in cancer, estimated to have about 50 % occurrence (Gasco et al., 2002; Marine et al., 2006; Haupt and Haupt, 2017). The percentage is even higher, when the inactivation in p53’s regulatory pathways is considered (Joerger and Fersht, 2016). Therefore, the use of cell lines which lack active p53, such as D492 and D492M, offers a different approach, more relevant for studying breast cancer signaling pathways, to study the role of DLK1-DIO3. The role of p53 in the cell is that of a tumor suppressor, impacting acts in proliferation, cell cycle and genomic stability (Mercer, 1992). In D492 cell lines, as could be expected, we did not observe effect on cell proliferation. Recently, Uroda and colleagues’ stated, that cell cycle arrest by *MEG3* is exclusively p53-dependent, (Uroda et al., 2019), in line with our suggestions that *MEG3* can have a different role in cells lacking p53. Collectively, these observations could explain the conflicting results about role of *MEG3* in tumors.

Many imprinted genes are located in clusters regulated by a differentially methylated regions (DMRs) (Bartolomei and Ferguson-Smith, 2011). In our study targeting the *MEG3* promoter, we have observed concomitant expression of *MEG3* with other miRNAs from the DLK1-DIO3 locus. Our data may support previous studies showing that the *MEG3* promoter controls expression of all maternally expressed genes from the DLK1-DIO3 locus (Tierling et al., 2006; Ioannides et al., 2014; Sanli et al., 2018). Zhu et al. (2019) have shown that the MEG3-DMR overlaps with the *MEG3* gene promoter and any deletion in this region inactivates both MEG3-DMR and the *MEG3* gene. Their data shows, that it is the MEG3-DMR, not the *MEG3* gene, which regulates imprinting (and expression). Therefore, by targeting the *MEG3* promoter at the MEG3-DMR all the non-coding RNAs at the DLK1-DIO3 locus are inactivated. *MEG3* expression can be considered as a marker for the expression of other ncRNAs at the locus.

Cellular plasticity, an important contributor to heterogeneity and drug resistance in breast cancer can be conveyed through EMT/MET (Liu et al., 2014). Partial EMT (p-EMT) may reflect cellular plasticity better than full-EMT and consequently, cells possessing this state adapt more easily to a new environment, which is necessary for cancer cell invasion and metastasis (Thiery, 2002; Tam and Weinberg, 2013; Lambert et al., 2017). Notably, a recent report highlights the importance of the intermediate stages of EMT for the intravasation of tumor cells and for metastasis formation in experimental breast or skin tumors (Pastushenko et al., 2018). Similarly, another study showed that cancer cells might only reach an intermediate EMT stage allowing for increased motility, while keeping its cellular plasticity (Brabletz et al., 2018). It has also been observed that full mesenchymal phenotype (EMT), has a low capacity to form metastasis compared to p-EMT (Schmidt et al., 2015). The essential criteria for aggressive behavior does not need to be a particular phenotype, but rather enhanced cellular plasticity, as is also observed for hybrid E/M cells (Grosse-Wilde et al., 2015). Thus, EMT may be viewed as a trans-differentiation process where epithelial and mesenchymal cells interconvert by passing through an intermediate “stem-like” state (Grosse-Wilde et al., 2018).

EMT is a complex process and meta-analysis indicates that there are possibly different types of EMT (Liang et al., 2016). We have shown, that by manipulating *MEG3* expression, and thus changing the expression of the non-coding genes at the DLK1-DIO3 locus, the majority of these EMT related TFs are affected, indicating an important role of the ncRNAs the DLK1-DIO3 locus in the EMT process. One of the most typical hallmarks of EMT is downregulation of *CDH1* (E-cadherin) and epithelial-specific keratins (Peinado et al., 2007). Altered expression of *MEG3* does not lead to change of E-cadherin expression and therefore *MEG3* may have induced only a partial EMT phenotype. However, it has been shown, that cells with p-EMT phenotype display concomitant expression of epithelial and mesenchymal markers (Armstrong et al., 2011) and loss of E-cadherin is not a prerequisite for EMT (Hollestelle et al., 2013). Cells undergoing collective migration have hybrid EMT phenotype characterized by E-cadherin expression, which helps to maintain cell–cell contacts (Friedl et al., 2012; Aceto et al., 2015). Furthermore, we have shown that altered expression of *MEG3* revealed distinct luminal and myoepithelial marker expression. Increased expression of *KRT14* and decreased expression *KRT19* indicate increased myoepithelial differentiation, which has been connected to a partial EMT phenotype (Petersen et al., 2001). Study on collective migration revealed *KRT14* as a key regulator of metastasis (Cheung et al., 2016) and the same applied for collective invasion, which was facilitated by subpopulation of cells expressing *KRT14* (Cheung et al., 2013). The observed increase of myoepithelial/basal differentiation in cells with higher expression of *MEG3* was supported with altered expression of other markers such as *KRT5*, *TP63*, and *CDH3*.

A key characteristic defining breast stem cells is the ability to form of mammospheres (Dontu et al., 2003; Grosse-Wilde et al., 2015). Morel and colleagues confirmed that human mammary epithelial cells undergoing EMT exhibited better mammosphere-forming capabilities (Morel et al., 2008) and Shimono et al. have shown that mammosphere-forming activity is abrogated in both normal and malignant mammary stem cells when the EMT program is shut down (Shimono et al., 2009). In this study phenotypic differences upon altered MEG3 expression were more prominent in 3D than in 2D cell culture, where *MEG3* increased mammosphere formation ability and slightly decreases branching potential in 3D culture. Furthermore, we have shown increased expression of *ALDH1A3* and *ITGA6*, in cells with overexpression of *MEG3*, supporting role of *MEG3* in stemness.

We propose that increased expression of *MEG3*, and thus increased expression of the ncRNAs at the DLK1-DIO3 locus, in D492 leads to partial EMT phenotype/enhanced plasticity, seen by molecular changes with increased mesenchymal and myoepithelial/basal genes and increased migration and resistance to apoptosis. In contrast, the repression of *MEG3*, and the maternally imprinted ncRNAs, in D492M leads to decreased mesenchymal and basal gene expression and decreased migration and resistance to apoptosis. Nguyen-Ngoc et al. also demonstrated, that motility can occur in cells that retain an epithelial molecular signature (Nguyen-Ngoc et al., 2012). This supports our observation, that manipulation of *MEG3* expression did not affect the morphological phenotype, but rather affected the functional phenotype. These characteristic properties of cells undergoing EMT were originally proposed to occur in breast cancer by Mani and colleagues (Mani et al., 2008), showing that stem-like and p-EMT properties share many characteristics, such as increased migration, resistance and survival (Creighton et al., 2009; Armstrong et al., 2011; Hanahan and Weinberg, 2011).

Increased understanding of branching morphogenesis in the breast and the regulation of EMT and MET may hold the key for future development of methods and drugs that neutralize the invading properties of cancer cells. Currently, there is need for biomarkers to accurately monitor the EMT/MET process that may improve treatment. Prognostic value of *MEG3* in human malignancies remains controversial and requires further investigation. Our results and conflicting data from the literature suggest that *MEG3* has a complex role in breast tissue.

## Data Availability Statement

The RNAseq data for this article has been submitted to GEO, with the GEO accession number GSE142268, see here: https://www.ncbi.nlm.nih.gov/geo/query/acc.cgi?acc=GSE142268

## Ethics Statement

The studies involving human participants were reviewed and approved by Icelandic National Bioethics Committee VSN-13-057 and VSN-11-105-V2. The Icelandic Data Protection Commission (2001/523 and 2002/463) Landspitali Ethical Committee No. 35/2013. The patients/participants provided their written informed consent to participate in this study.

## Author Contributions

MM, TG, JB, ZB, EB, GT, and BH: conceptualization and design of the study. ZB, EB, JB, AS, and BH: data acquisition. ZB, AS, EB, GT, SS, and BH: data analysis. ZB, GT, TG, and BH: drafting the manuscript. All authors participated in data interpretation, revision of the manuscript and approved the final version to be published.

## Conflict of Interest

The authors declare that the research was conducted in the absence of any commercial or financial relationships that could be construed as a potential conflict of interest.

## Footnotes

1 http://co.bmc.lu.se/gobo/
