# Supplementary material for: Expression of ncRNAs on the DLK1-DIO3 Locus Is Associated With Basal and Mesenchymal Phenotype in Breast Epithelial Progenitor Cells
Source: Front Cell Dev Biol. 2020 Jun 16;8:461. doi: 10.3389/fcell.2020.00461 (PMC7308478; doi:10.3389/fcell.2020.00461)
Supplement: Supplementary file 1 [file Data_Sheet_1.pdf]

Supplementary material

Antibodies for western blot: CDH1 (E-cadherin, BD Transduction Labs, 610182), CDH2 (N-cadherin, BD Transduction Labs, 610921)

Primers for qRT-PCR: *ALDH1A3* (IDT, Hs.PT.56a.657970), *INTGA6* (IDT,Hs.PT.58.453862).

Supplementary methods

Cell proliferation assay

Cell proliferation assay was performed on IncuCyte Zoom microscope (Essen Bioscience) according to manufacturer´s protocol. Cells were plated at density 10 x 10^3/ well in 96 - well plate (Corning, 353072). Cell culture media was changed three times per week.

Cell invasion assay

Cell invasion was examined using transwell filters with 8 μm pore size (Corning, 353097) with a layer of Matrigel, diluted 1:10 with H14 media. Briefly, 3 x 10^3 cells were resuspended in 250 μl H14 medium and placed on top of Matrigel and 500 μl of H14 + 10% FBS was added to the lower chambers, bellow filter. Cell were incubated for 48 hours in 5% CO_2 at 37°C. After incubation, the Matrigel was removed as well as non-invasive cells from the upper part of the filter with cotton swab and washed in between 3 x with 1 x PBS. The filters were then fixed with methanol and stained with DAPI. Cell were photographed in three random fields. Pictures were analysed wit ImageJ Software.

Scratch wound assay

The assay was performed according to manufacturer's protocol. 6 x 10^3 cells/ well were seeded so they were 100% confluent following day into ImageLock Plates (Essen Bioscience, 4379). Woundmaker (Essen Bioscience, 4493) was used to do the scratch. The images were taken every two hours. Images were analysed as *Relative Wound Density (%)*.

Statistical analysis

Statistical differences of qRT-PCRs (Supplementary Figure 6) and functional assay (Supplementary Figure 5B) between samples were assessed with paired Student t-test. Statistical differences in Supplementary Figure 5A and 5C was calculated using multiple unpaired Student t-test per row. Statistical differences of quantifications of western blots (Supplementary Figure 4) among samples were assessed using one- way ordinary ANOVA, followed by Tukey´s multiple comparison test. *P* values below 0.05 were considered significant (*$p \leq 0.05$; **$p \leq 0.01$; ***$p \leq 0.001$; ****$p \leq 0.0001$). All statistical analysis was performed in GraphPad Prism.

Supplementary Figures

Name	Fold change	P-value
mir-370	4,49	0.0016
mir-1185-2	3,97	0.0504
mir-770	3,95	0.0166
mir-376a-2	3,74	0.0612
mir-494	3,61	0.0032
mir-382	3,46	0.0144
mir-654	3,23	0.0054
mir-541	3,20	0.2823
mir-337	3,18	0.0085
mir-485	2,80	0.0139
mir-299	2,77	0.0172
mir-1185-1	2,56	0.0547
mir-377	2,56	0.0398
mir-127	2,53	0.0307
mir-376a-1	2,47	0.0475
mir-758	2,33	0.0549
mir-493	2,32	0.0374
mir-487b	2,31	0.0563
mir-369	2,18	0.0640
mir-432	2,17	0.0738
mir-154	2,13	0.0954
mir-543	2,10	0.1642
mir-134	2,09	0.2873
mir-656	2,03	0.1442
mir-412	1,96	0.1800
mir-379	1,93	0.1593
mir-411	1,85	0.1140
mir-668	1,82	0.2650
mir-380	1,70	0.3259
mir-431	1,69	0.3057
mir-376c	1,65	0.2135
mir-381	1,55	0.5539
mir-323b	1,54	0.3447
mir-495	1,34	0.5132
mir-433	1,31	0.5713
mir-487a	1,30	0.6391
mir-655	1,28	0.5625
mir-655	1,28	0.5625
mir-410	1,26	0.5627
mir-1197	1,26	0.6935
mir-539	1,16	0.7161
mir-496	1,08	0.8971
mir-376b	1,06	0.8880
mir-136	1,01	0.8938
mir-409	-1,37	0.6847
mir-323	#N/A	#N/A
mir-329-1	#N/A	#N/A
mir-329-2	#N/A	#N/A
mir-300	#N/A	#N/A
mir-889	#N/A	#N/A
mir-544	#N/A	#N/A

Supplementary Figure 1. The ncRNAs from DLK1-DIO3 locus have increased expression in mesenchymal cells. Majority of the miRNAs from the DLK1-DIO3 locus are upregulated in D492M compared to D492. Small RNA sequencing: the list of miRNAs from the DLK1-DIO3 locus, where majority of them are upregulated in D492M.

(A)

no	term	count	%	p-value
1	Extracelllar matrix	26	1.45903	1.56E-14
2	Proteinaceous extracellular matrix	25	1.40292	2.62E-14
3	Cell adhesion	35	1.96409	1.33E-13
4	Cell attachment site	15	0.84175	1.33E-13
5	Biological adhesion	35	1.96409	1.38E-13
6	Extracelllar matrix	21	1.17845	2.06E-13
7	Extracelllar region part	38	2.13244	1.63E-12
8	Cell adhesion	25	1.40292	2.77E-12
9	Signal	71	3.98429	6.65E-12
10	Signal peptide	71	3.98429	8.99E-12
11	EGF-like domain	19	1.06622	9.40E-12
12	AGF calcium-binding	13	0.72952	1.10E-11
13	Extracellular region	54	3.0303	2.07E-11
14	EGF-like, type 3	18	1.0101	2.25E-11
15	EGF-like region, conserved site	21	1.17845	3.50E-11

(B)

Spearman correlation with *MEG3*				
	Normal breast		Breast cancer	
Mesenchymal markers	rho	p-val	rho	p-val
ACTA2	0.098	7.64E-02	0.526	1.03E-80
AXL	0.196	3.31E-04	0.455	3.36E-58
CD61 (ITGB3)	0.288	9.45E-08	0.284	3.18E-22
CDH2	0	9.99E-01	0.156	1.57E-07
FOSL2	0.451	4.97E-18	0.249	2.95E-17
FOXC1	0.138	1.20E-02	0.121	5.09E-05
FOXC2	0.304	1.57E-08	0.3	1.11E-24
LAMA1	0.236	1.42E-05	0.467	8.03E-62
LOX	0.165	2.55E-03	0.398	1.10E-43
MMP2	0.45	6.60E-18	0.656	9.34E-139
SMAD4	0.091	9.86E-02	0.017	5.81E-01
SNAI1	0.169	2.09E-03	0.163	3.82E-08
SNAI2	0.092	9.65E-02	0.469	2.01E-62
TGFB1	0.628	1.10E-37	0.508	2.59E-74
TWIST1	0.405	1.71E-14	0.516	3.14E-77
TWIST2	0.357	2.20E-11	0.636	3.81E-128
VIM	0.01	8.54E-01	0.437	2.23E-53
YAP1	-0.287	1.07E-07	0.191	1.28E-10
ZEB1	0.2	2.45E-04	0.498	3.28E-71
ZEB2	0.277	3.89E-07	0.462	3.68E-60

Supplementary Figure 2. *MEG3* correlates with expression of mesenchymal genes. **(A)** *MEG3* correlates with expression of extracellular matrix genes. MEG3 correlated pathway analysis (GOBO). **(B)** *MEG3* correlates with expression of mesenchymal genes in normal breast and breast cancer tissue. Listed mesenchymal genes shows positive correlation with *MEG3* in MiPanda dataset. The Spearman correlation above 0.3 is considered as fair positive correlation are highlighted in orange.

(A)

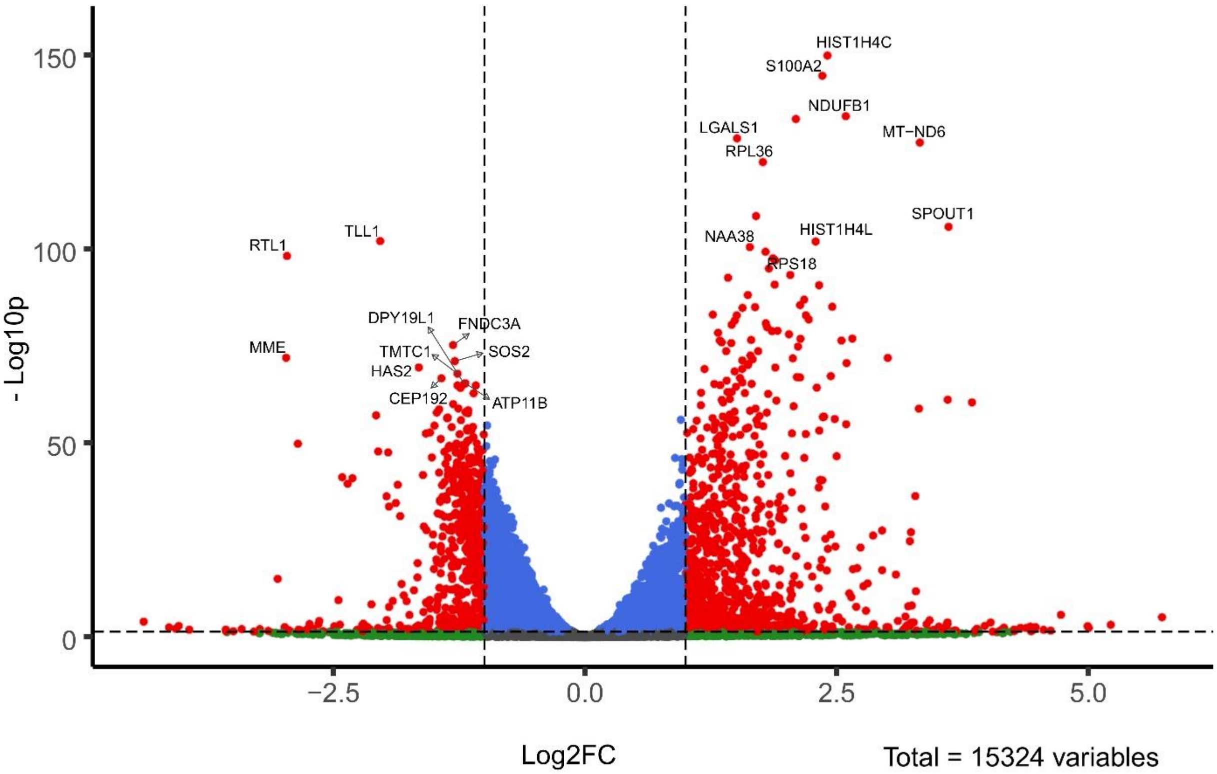

HIST1H4C
S100A2
NDUFB1
LGALS1
MT−ND6
RPL36
SPOUT1
TLL1
RTL1
NAA38
HIST1H4L
RPS18
DPY19L1
FNDC3A
MME
TMTC1
SOS2
HAS2
CEP192
ATP11B
150
100
50
0
- Log10p
−2.5
0.0
2.5
5.0
Log2FC
Total = 15324 variables

(B)

Top 30 downregulated genes in D492M$^{KD-MEG3}$

Gene	Log2 FC	p value
AC011005.1	-1.91	5.80E-10
ADGRL3	-1.77	6.60E-10
ADGRL4	-3.05	1.20E-15
CFH	-2.07	8.20E-58
CLDN24	-1.97	5.10E-05
CSMD3	-2.2	4.20E-04
DCT	-2.43	8.10E-04
EMB	-2.05	1.70E-48
GALNT3	-2.85	1.80E-50
GRIK2	-2.64	8.60E-05
HOOK1	-1.95	2.10E-08
HS6ST3	-4.38	1.30E-04
LRRCC1	-1.8	2.10E-11
MME	-2.97	1.20E-72
PKHD1L1	-2.12	4.40E-09
PKIA	-2.36	3.50E-40
POSTN	-2.45	3.70E-10
POTEE	-2.31	1.30E-41
POTEJ	-2.41	7.40E-42
RASEF	-1.86	6.70E-40
RGPD2	-1.82	2.40E-14
RGPD3	-1.88	3.30E-35
RTL1	-2.96	6.70E-99
SETSIP	-1.97	6.20E-37
SULF1	-1.96	3.00E-48
TLL1	-2.03	9.90E-103
ZC3H11B	-1.84	8.00E-32
ZDBF2	-2.74	5.10E-04
ZFY	-2.65	5.40E-04
ZNF711	-1.95	2.70E-34

Top 30 upregulated genes in D492M$^{KD-MEG3}$

Gene	Log2 FC	p value
APOA1	3.28	5.40E-37
AZU1	3.24	2.70E-04
C1orf232	2.87	5.60E-04
C1QTNF5	3.11	8.30E-05
CLDND2	3.32	1.50E-59
FFAR1	3.42	5.90E-05
FOXN1	2.95	6.80E-18
GNG8	3.21	1.40E-08
GSG1L2	3.6	2.00E-04
GUCY1A2	3.24	1.10E-27
HBZ	3.09	9.30E-17
HCRT	3.27	2.00E-04
HRC	2.86	8.10E-27
IL13RA2	3.18	6.20E-06
IYD	3.6	7.40E-04
MT-ND3	3.01	1.30E-72
MT-ND6	3.33	3.90E-128
MTRNR2L1	5.22	8.40E-04
MTRNR2L12	3.29	1.90E-12
NRXN1	4.73	2.20E-06
OR5AU1	3.39	6.10E-04
POU2F3	3.84	3.80E-61
PSORS1C2	3.23	2.30E-25
RPS29	2.95	4.00E-28
S100A8	3.25	8.90E-09
SPINK1	4.03	2.00E-04
SPOUT1	3.61	2.00E-106
TCF23	3.6	6.10E-04
TSPAN19	3.6	7.60E-62
UPK3BL1	5.73	8.90E-06

Supplementary Figure 3. RNA-sequencing analysis of D492M$^{KD-MEG3}$ vs. D492M$^{KD-MEG3.}$ **(A)** Volcano plot over all data ($q < 0.05$, TPM > 1) showing symmetric distribution of the RNA-sequencing data. The top ten downregulated and top ten upregulated genes according log2 fold change in D492M$^{KD-MEG3}$ are labelled with gene names. **(B)** The table with top 30 downregulated genes in D492M$^{KD-MEG3}$ (left) and top 30, upregulated genes in D492M$^{KD-MEG3}$ (right).

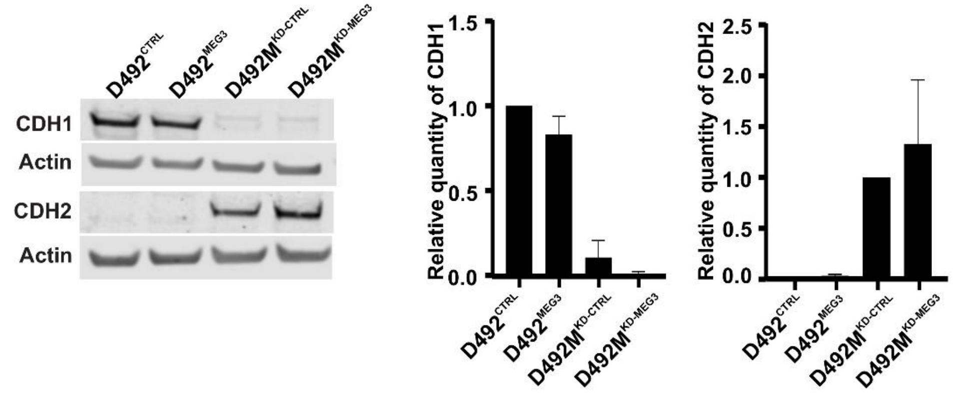

Supplementary Figure 4. *MEG3* does not change expression of CDH1 and CDH2. Representative pictures of western blot (WB) with its quantification bellow. $D492^{MEG3}$ has slightly decreased CDH1 (E-cad) on protein level compared to $D492^{CTRL}$. $D492M^{KD\text{-}MEG3}$ has slightly increased CDH2 (N-cad) on protein level compared to $D492M^{KD\text{-}CTRL}$, however with no statistical significance WB results shown as mean ± SD. One-way ordinary ANOVA followed by Tukey´s multiple comparison test was used to test significance; n = 3.

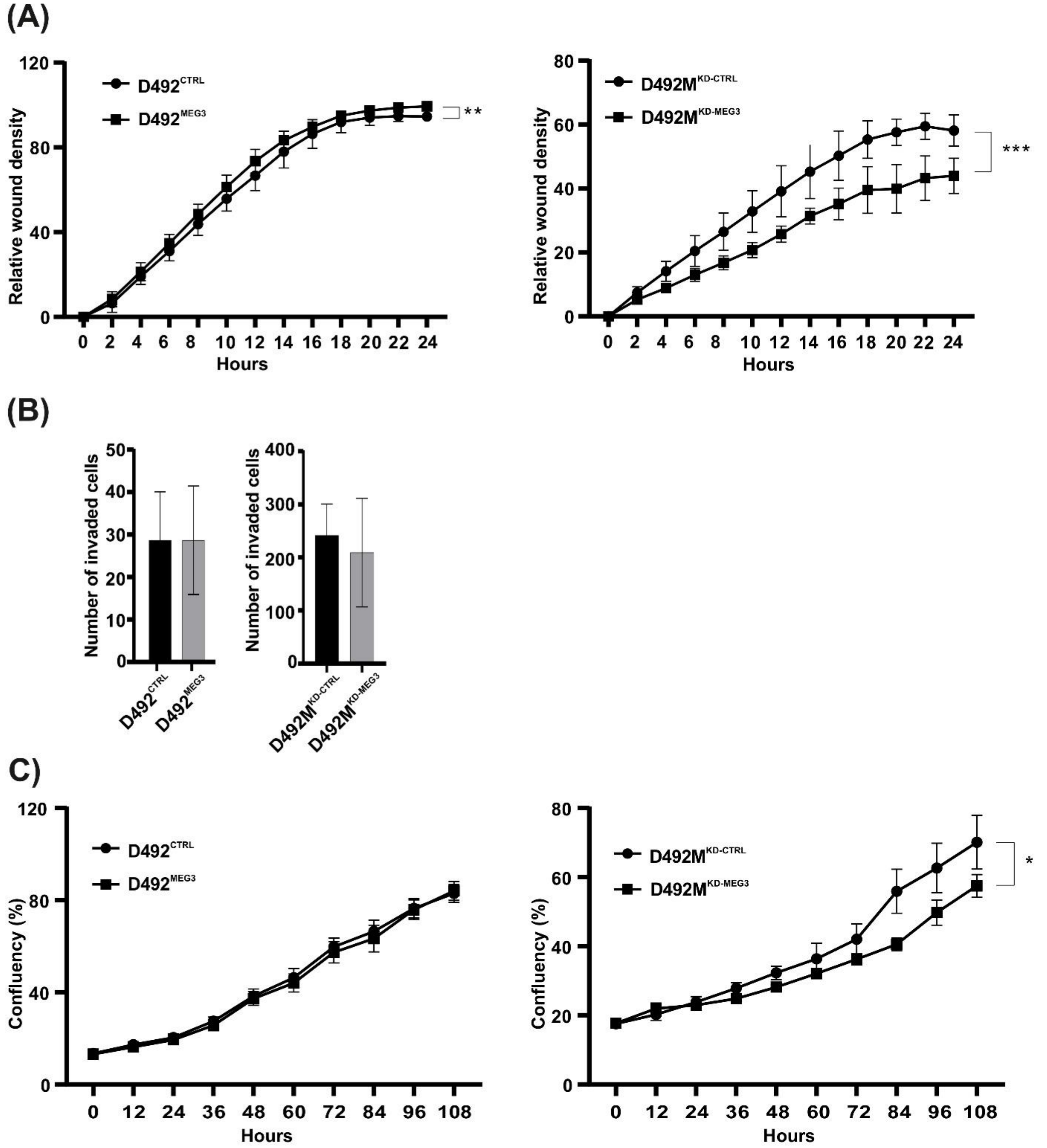

Supplementary Figure 5. *MEG3* does not influence invasion but has effect on wound healing migration. **(A)** *MEG3* slightly increase wound healing migration and knock-down of *MEG3* decrease wound healing migration. D492^{MEG3} has slightly increased migration rate compared to D492^{CTRL} (left). D492M$^{KD\text{-}MEG3}$ migrates less compared to D492M$^{KD\text{-}CTRL}$ (right). Data is analysed on Incucyte Zoom and results displayed as Relative wound density with mean ± SD. Multiple unpaired Student t-test per row was used to test significance, n = 6 ; ** $p \leq 0.01$; *** $p \leq 0.001$; with statistical differences at 24-hour timepoint. **(B)** *MEG3* and knock-down of *MEG3* does not have effect on invasion. D492^{MEG3} has comparable invasion rate with D492^{CTRL} (left). As well as D492M$^{KD\text{-}MEG3}$ has comparable invasion rate with D492M$^{KD\text{-}CTRL}$ (right). Quantification of number

of invaded cells shown as mean ± SD. Unpaired t-test was used to test significance, n = 6. **(C)** *MEG3* does not have effect on proliferation, while knock-down of *MEG3* has. Proliferation assay: D492^{MEG3} has comparable proliferation rate with D492^{CTRL} (left). D492M$^{KD\text{-}MEG3}$ slightly reduced proliferation rate compared to D492M$^{KD\text{-}CTRL}$ (right). Data is analysed on Incucyte Zoom and results displayed as Confluency percentage as mean ± SD. Multiple unpaired Student t-test per row was used to test significance at 24-hour time point; n = 6; *$p \leq 0.05$.

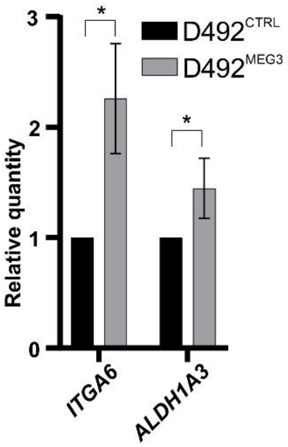

Supplementary Figure 6. *MEG3* increases expression of stem cell marker *ITGa6* and *ALDH1A3*. qRT-PCR showing D492^{MEG3} has increased expression of *ITGa6* (Integrin alpha 6) and *ALDH1A3* (Aldehyde dehydrogenase) compared to D492^{CTRL}. Results shown as mean ± SD. Unpaired t-test was used to test significance: * $p \leq 0.05$; n = 3.
